# DFT supported computational insights into Withaferin A structure and its binding interaction with Hsp90

**DOI:** 10.1038/s41598-025-33145-w

**Published:** 2025-12-29

**Authors:** Romash Shoni, Phadindra Raj Karki, Khagendra Tripathi

**Affiliations:** Department of Physics, St. Xavier’s College, Tribhuvan University, Kathmandu, Nepal

**Keywords:** Biochemistry, Chemical biology, Chemistry, Computational biology and bioinformatics, Drug discovery

## Abstract

Withaferin A, a bioactive steroidal lactone, was systematically investigated using density functional theory (DFT) and complementary computational approaches to understand its structural stability, electronic reactivity, and therapeutic potential. The combined spectroscopic and electronic analyses confirmed strong electron delocalization, stable molecular configuration, and favorable charge-transfer characteristics. Natural bond orbital and frontier molecular orbital studies revealed extensive hyperconjugation and high kinetic stability, while thermodynamic assessments indicated spontaneous and stable behavior across varying temperatures. Molecular docking with Hsp90 demonstrated strong binding affinity, stabilized by hydrogen bonding and Van der Waals interactions with key residues. These findings highlight Withaferin A’s structural robustness, electronic versatility, and potent binding characteristics, supporting its promise as a therapeutic inhibitor. Overall, this integrative computational study provides valuable molecular-level insights that can guide future experimental validation and rational drug design involving Hsp90-targeted compounds.

## Introduction

Withaferin A, a bioactive steroidal lactone derived from *Withania somnifera* (Ashwagandha), has garnered considerable attention for its diverse pharmacological properties, including anticancer, anti-inflammatory, neuroprotective, and cardioprotective activities^1–7^. Its anticancer potential is particularly notable, as Withaferin A modulates multiple cellular pathways involved in tumor growth and progression, including apoptosis, oxidative stress, autophagy, and regulation of heat shock proteins (HSPs)^4,8^. Studies have demonstrated activity against various cancers such as ovarian, breast, lung, prostate, and colorectal cancers^9–11^, and its combination with chemotherapeutic agents such as doxorubicin, oxaliplatin, and paclitaxel or with radiation therapy enhances treatment efficacy by promoting apoptosis through caspase-3/PARP activation and inhibiting pro-survival mechanisms^8^. These reports highlight its promise as both a chemotherapeutic and a radiosensitizing agent.

Computational techniques provide complementary insights into molecular behavior. Density functional theory (DFT) enables accurate prediction of molecular geometry, vibrational spectra, and electronic properties, facilitating the understanding of reactivity and stability^12^. Complementarily, molecular docking serves as an essential technique in structure-based drug design, allowing prediction of ligand protein interactions and binding conformations, guiding structure-based drug^13–15^.

Despite extensive biological studies, no integrated computational investigation of Withaferin A has comprehensively explored its structural, vibrational, electronic, and biological interaction properties. Previous work has largely focused on pharmacological and cellular effects^16,17^, limiting mechanistic understanding at the molecular level. This gap restricts the deeper understanding necessary for optimizing its therapeutic potential and guiding the design of more effective derivatives.

**Fig. 1:**
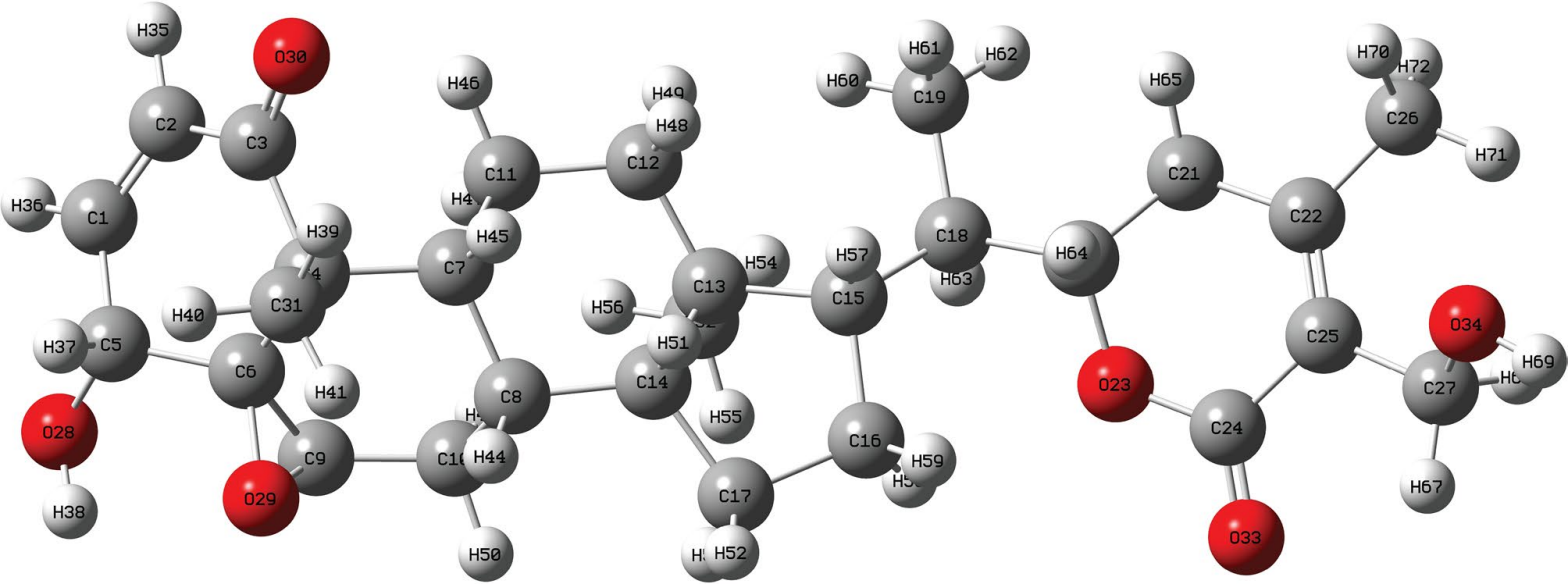
Geometry-optimized structure of Withaferin A displaying atom-wise labeling.

To address this gap, the present study employs DFT with the B3LYP functional^18–20^ to analyze the structural, electronic, and vibrational properties of Withaferin A (Fig. 1), complemented by molecular docking with Hsp90. This integrated approach provides detailed molecular-level insights, supporting its anticancer potential and guiding future experimental and therapeutic development .

## Methodology

All quantum chemical calculations were performed using the B3LYP functional with the 6-311G(d) basis set within Density Functional Theory (DFT) using Gaussian 09W software^21^. The computational study of Withaferin A included geometry optimization, vibrational frequency predictions, frontier molecular orbitals (FMOs), $^1$H and $^{13}$C NMR spectra, Natural Bond Orbital (NBO) analysis, Molecular Electrostatic Potential (MEP), Mulliken atomic charges, thermodynamic parameters, Fukui functions, and contour plots. Reduced density gradient (RDG), electron localization function (ELF), and localized orbital locator (LOL) analyses were performed using Multiwfn software^22^, while the Density of States (DOS) was evaluated using GaussSum^23^. Potential Energy Distribution (PED) analysis was carried out using VEDA4 software^24^. Molecular docking studies were performed using AutoDock Vina^25^ to assess binding interactions with Hsp90. Python and Origin software^26^ were employed for data visualization.

## Results and discussions

### Vibrational analysis (IR and Raman)

**Fig. 2:**
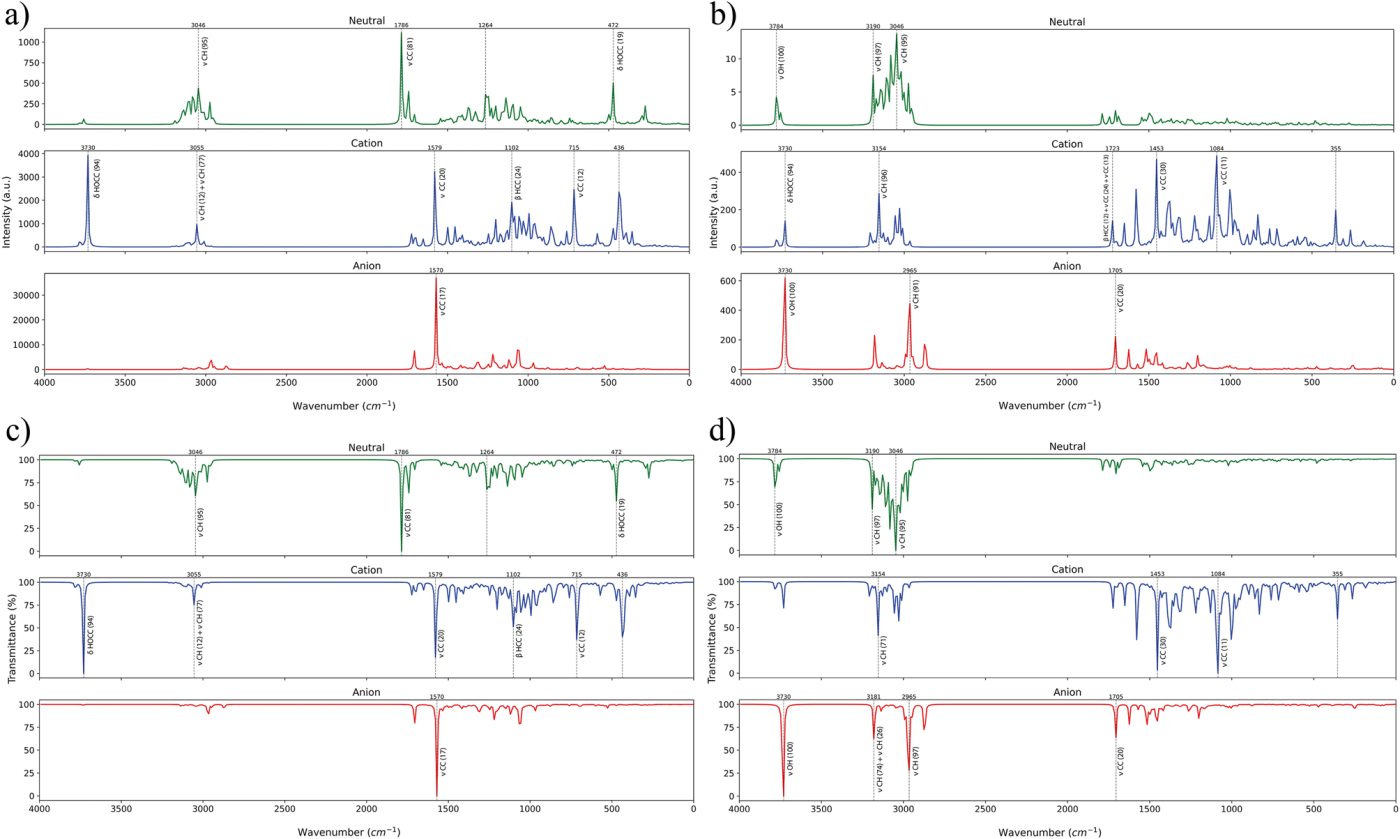
Vibrational spectroscopy of Withaferin A. **a** IR spectrum, **b** Raman spectrum, **c** FT-IR spectrum, and **d** FT-Raman spectrum. Here, $\nu$, $\beta$, and $\delta$ represent stretching, bending, and torsional vibrations, respectively. Vibrational modes with less than 10% PED contribution are not assigned.

Vibrational frequencies, which describe the periodic motion of atoms in a molecule, typically occur between 300 $\hbox {cm}^{-1}$ and 3000 $\hbox {cm}^{-1}$^27,28^. The IR and Raman spectra of Withaferin A exhibit distinct peaks at characteristic wavenumbers, enabling the identification of functional groups^29^. The vibrational spectra of Withaferin A in its neutral, cation, and anion states were computed in the gas phase using the B3LYP/6-311G(d) level of theory. Withaferin A, composed of 72 atoms, possesses 210 fundamental vibrational modes. The harmonic frequencies were scaled using established correction factors^30^, and mode assignments were carried out with the help of potential energy distribution (PED) analysis, confirming the contributions of atomic displacements to individual vibrational motions. Figure 2 presents the vibrational spectra of Withaferin A in all three charge states, showing distinct peaks corresponding to stretching, bending and torsion vibrations. Complete PED analyses for all vibrational modes are provided in Supplementary Tables S1–S3.

Aromatic C–H stretching vibrations were consistently observed within the expected 3100–3000 $\hbox {cm}^{-1}$ range^31–35^, and their characteristic spectral patterns enable straightforward identification. In the present study, all C–H stretching modes (mode numbers 210–169) fell within this range, confirming typical aromatic behavior^27,32,33,35^. For the neutral molecule, the bands appeared between 3086.47 $\hbox {cm}^{-1}$ and 2855.45 $\hbox {cm}^{-1}$, while in the cation and anion states they were observed at 3099.81–2869.58 $\hbox {cm}^{-1}$ and 3074.53– 2776.88 $\hbox {cm}^{-1}$, respectively. PED values of 80–100% across these modes indicate strong vibrational contributions. Notably, the Raman spectra exhibited more intense C–H stretching features than the IR spectra, reflecting Raman’s greater sensitivity to these vibrations. The experimental IR (KBr) spectrum^36^ exhibited characteristic absorption bands, which are well reproduced by our B3LYP/6-311G(d) calculations, confirming the overall consistency between experiment and our study. In addition, aromatic C=C ring stretching bands were identified in the 1280–1380, 1430–1465, 1470–1540, 1575–1590, and 1590–1625 $\hbox {cm}^{-1}$ regions, with intensities varying according to the molecular environment^35,37^. These features were observed across all charge states with significant PED involvement. C O stretching vibrations appeared within the 1260–1000 $\hbox {cm}^{-1}$ interval. H C C bending vibrations were detected between 1452.81 $\hbox {cm}^{-1}$ and 1219.53 $\hbox {cm}^{-1}$, exhibiting weak PED contributions, whereas H C C C torsional modes were identified between 1489.45 $\hbox {cm}^{-1}$ and 1440.15 $\hbox {cm}^{-1}$, along with additional lower-frequency torsions.

### NMR spectroscopy

NMR spectroscopy is a widely used and powerful technique for detailed structural characterization of organic compounds. It provides insights into both intra- and intermolecular hydrogen bonding^38^ and relies on the response of isotropic chemical shielding (ICS) to the local chemical environment of the nucleus^39^. This method enables the identification of ionic species and functional groups while offering magnetic properties that support accurate predictions of molecular geometries^35^.

**Fig. 3:**
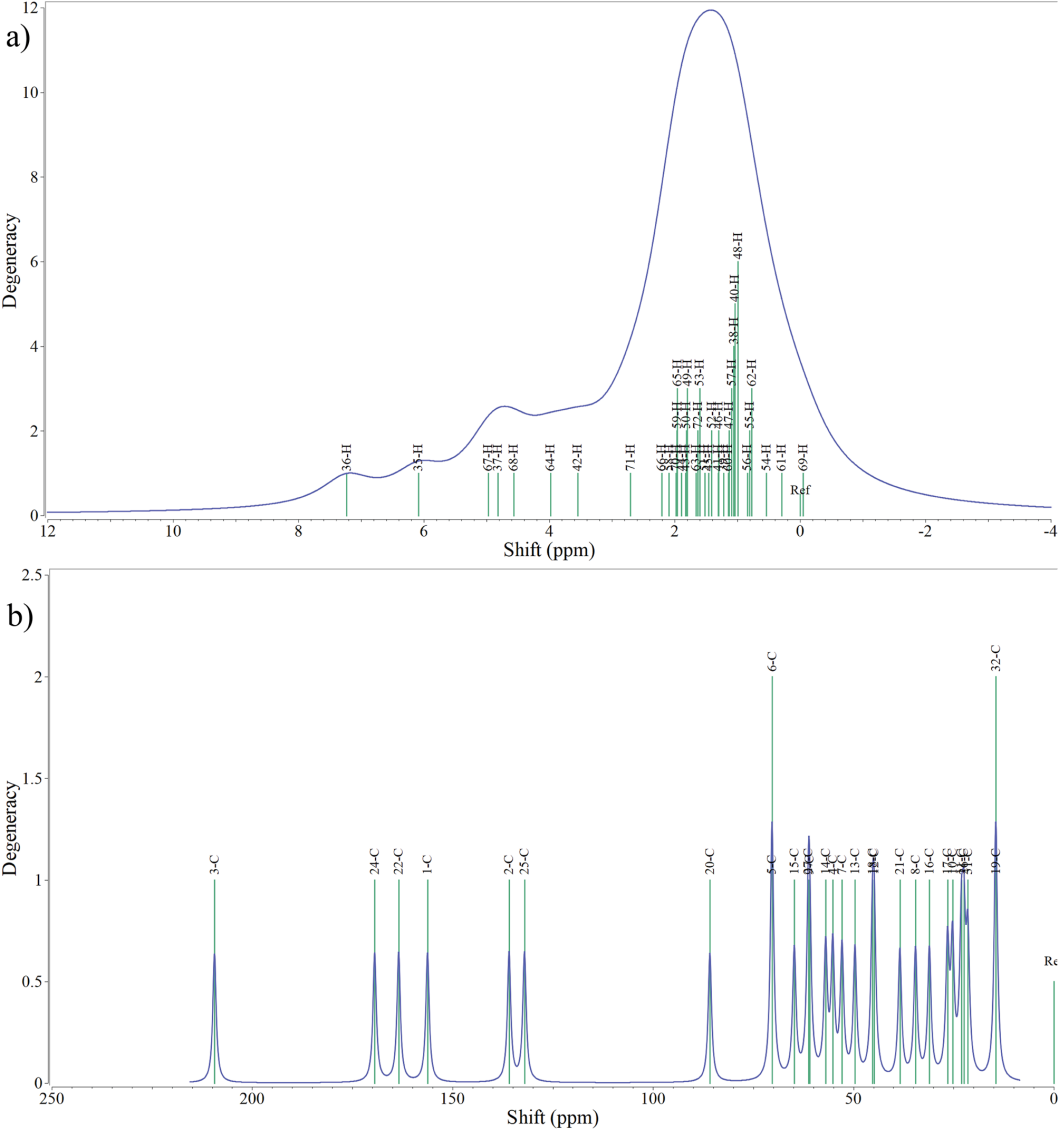
NMR spectra of Withaferin A in the neutral state: **a** H-NMR spectrum and **b** C-NMR spectrum.

For Withaferin A, $^{1}$H and $^{13}$C NMR chemical shifts of the neutral molecule were calculated using the GIAO-SCF method at the B3LYP/6-311+G(2d,p) level of theory, with tetramethylsilane (TMS) as the reference. The referenced shielding constants were 31.8821 ppm for $^{1}$H and 182.466 ppm for $^{13}$C. The resulting theoretical $^1$H and $^{13}$C chemical shifts are presented in Fig. 3a and b, respectively, showing a broad range of signals. The $^1$H chemical shifts range from ${-0.0471}$ ppm (69H) to 7.2307 ppm (36H), reflecting a broad spectrum of proton environments within the molecule. The lower chemical shifts correspond to highly shielded aliphatic protons, while the deshielded resonance near 7.23 ppm originates from a vinylic hydrogen associated with an unsaturated region of the molecule. The $^{13}$C chemical shifts extend from 14.4718 ppm (32C) to 209.3942 ppm (3C), indicating considerable variation in carbon electronic environments. The most deshielded carbon signal at 209.3942 ppm is characteristic of a ketone carbonyl, confirming the presence of a ketone functionality. Additional resonances at 169.4373 ppm (24C) and 163.4553 ppm (22C) arise from conjugated and lactone carbonyl carbons. Carbons resonating between 132.06 ppm and 156.26 ppm are olefinic $\hbox {sp}^{2}$ carbons involved in conjugation. The calculated $^{1}$H and $^{13}$C NMR chemical shifts collectively validate the existence of ketone, lactone, hydroxyl, and conjugated double-bond functionalities, fully consistent with the established structure and reactivity of Withaferin A.

### Natural Bond Orbital (NBO) analysis

Natural Bond Orbital (NBO) analysis identifies bonding orbitals with the highest electron density among atoms^35,40,41^. This method provides insights into interactions between occupied and virtual orbitals, thereby improving the understanding of inter- and intramolecular interactions^32^. In this study, the second-order Fock matrix was employed to quantitatively evaluate donor-acceptor interactions. The stabilization energy, $E^{(2)}$, corresponding to the delocalization from a donor (i) to an acceptor (j), is expressed as:

1 $$\begin{aligned} E^{(2)} = \Delta E_{ij} = \frac{q_i[F(i,j)]^2}{(E_j - E_i)} \quad \text { 35} \end{aligned}$$

where $q_i$ represents the occupancy of the donor orbital, $E_i$ and $E_j$ are the diagonal elements, and *F*(*i*, *j*) denotes the off-diagonal element of the NBO Fock matrix^35^. The second-order perturbation energies ($E^{(2)}$, in kcal/mol), representing stabilization or interaction energies, were calculated at the DFT/B3LYP/6-311G(d) level^42^. A higher stabilization energy indicates stronger donor-acceptor interactions. In general, donor orbitals include $\sigma$ and $\pi$ types, while acceptor orbitals consist of $\sigma ^*$ and $\pi ^*$ types^43^. These interactions are denoted as BD(1) = $\sigma$, BD(2) = $\pi$, BD*(1) = $\sigma ^*$, and BD*(2) = $\pi ^*$. For the neutral state, NBO analysis revealed strong hyper-conjugative interactions with stabilization energies such as: BD*(2)C24–O33 $\rightarrow$ BD*(2)C22–C25 [$E^{(2)} = 61.08$ kcal/mol], LP(2)O23 $\rightarrow$ BD*(2)C24–O33 [$E^{(2)} = 34.66$ kcal/mol], and LP(2)O33 $\rightarrow$ BD*(1)O23–C24 [$E^{(2)} = 31.7$ kcal/mol]. These results indicate extensive electron delocalization, enhancing molecular stability. In the cation state, notable interactions include: BD*(2)C24–O33 $\rightarrow$ BD*(2)C22–C25 [$E^{(2)}= 37.32$ kcal/mol] and LP(2)O23 $\rightarrow$ BD*(2)C24–$\hbox {O}_{33}$ [$E^{(2)}= 18.8$ kcal/mol]. For the anion state, the most significant interactions were: LP(2)O33 $\rightarrow$ BD*(1)O23-C24 [$E^{(2)} = 15.7$ kcal/mol] and LP(1)C3 $\rightarrow$ BD*(2)C1–C2 [$E^{(2)} = 47.44$ kcal/mol], with the former representing the highest stabilization energy observed across all charge states.A unusually high stabilization energy was observed: BD*(2)C24 O33 $\rightarrow$ BD*(2)C22 C25, measuring 167.17 kcal/mol in the anion state. This is likely a artifact of the computational method which require additional computational or experimental validation.

Overall, these findings highlight the presence of strong hyper-conjugative interactions and significant electron delocalization, contributing to the stability of Withaferin A in different states and supporting the occurrence of intra-molecular charge transfer. However, some unusually high stabilization energies may stem from approximation errors, underscoring the need for experimental validation. Detailed parameters, including intra-molecular donor–acceptor interactions and stabilization energies for selected bonds in the neutral, cation, and anion states, are provided in Supplementary Tables S4–S6.

### UV-vis absorption analysis

The charge transfer characteristics of organic compounds can be effectively examined through UV–Vis absorption spectra, as these reflect underlying electronic excitation processes^43^. According to the Franck–Condon principle, the absorption maximum ($\lambda _{\text {max}}$) corresponds to a vertical electronic transition, typically involving excitation of an electron from the HOMO to the LUMO and marking the shift from the ground to the first excited state^35,44^.

**Fig. 4:**
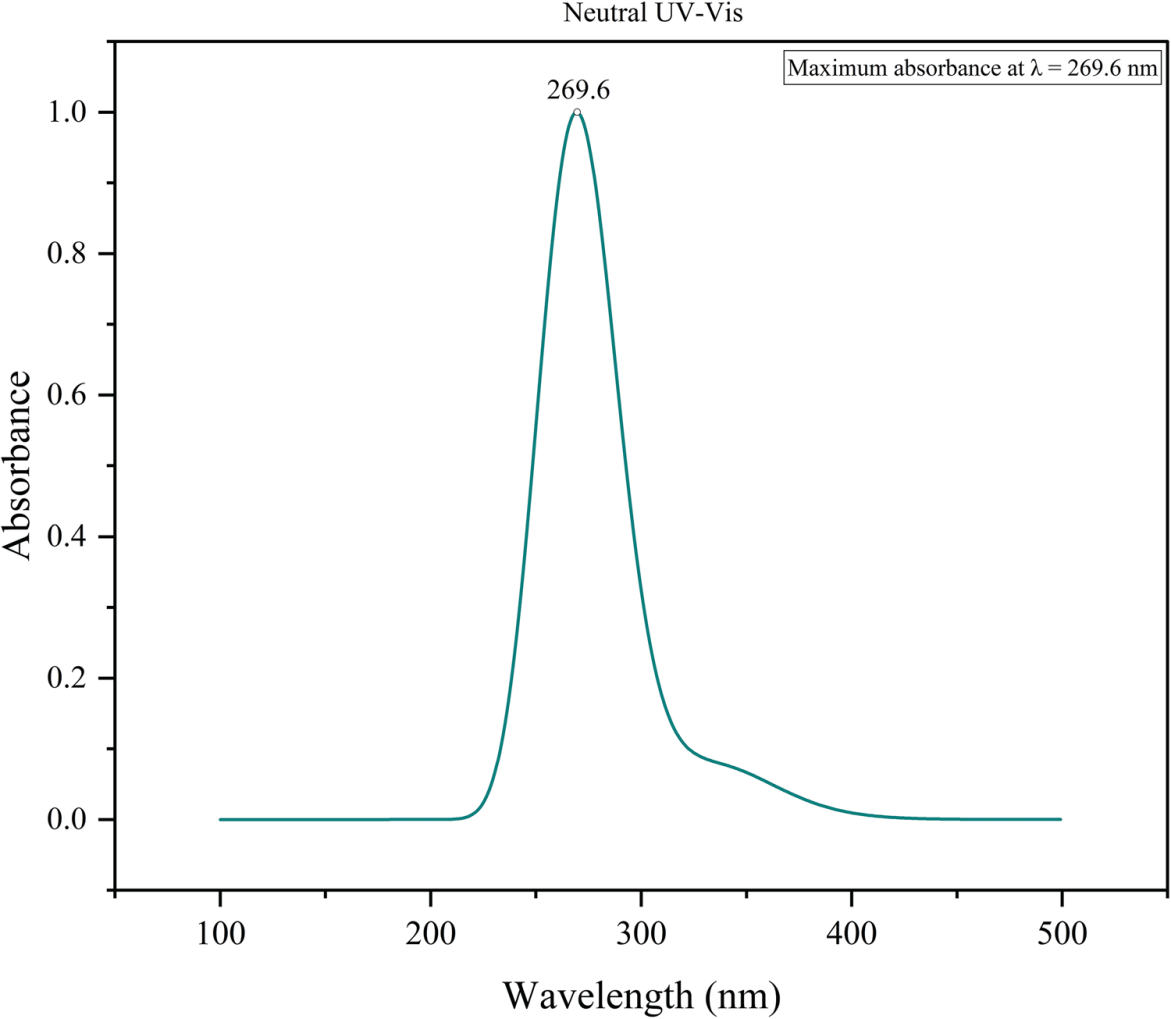
UV Vis absorption spectrum of Withaferin A.

In the present study, the lowest-energy electronic transition for the neutral state of Withaferin A was calculated at 337.94 nm with an oscillator strength (*f*) of 0.002, indicating a very weak transition. TD–DFT analysis further revealed that this excitation arises predominantly from the HOMO $\rightarrow$ LUMO configuration, contributing 95.13% to the overall transition. The oscillator strength (*f*), a dimensionless parameter, quantifies the intensity of such electronic excitations^45^. Although the first electronic excitation occurs at 337.94 nm, the simulated UV–Vis spectrum shows the most intense absorption band at 269.6 nm for the neutral state (Fig. 4), corresponding to higher-energy transitions with greater oscillator strengths. These peaks represent the cumulative effect of multiple electronic transitions within the molecule. While the discussion here focuses on the neutral form, the spectral characteristics of the cationic and anionic states are summarized in Supplementary Table S7. Furthermore, the excitation wavelengths ($\lambda$), oscillator strengths (*f*), and excitation energies (eV) for each charge state were computed at the TD–DFT/B3LYP/6-311G(d) level, providing a comprehensive picture of the electronic excitation behavior of Withaferin A.

### ELF and LOL analysis

**Fig. 5:**
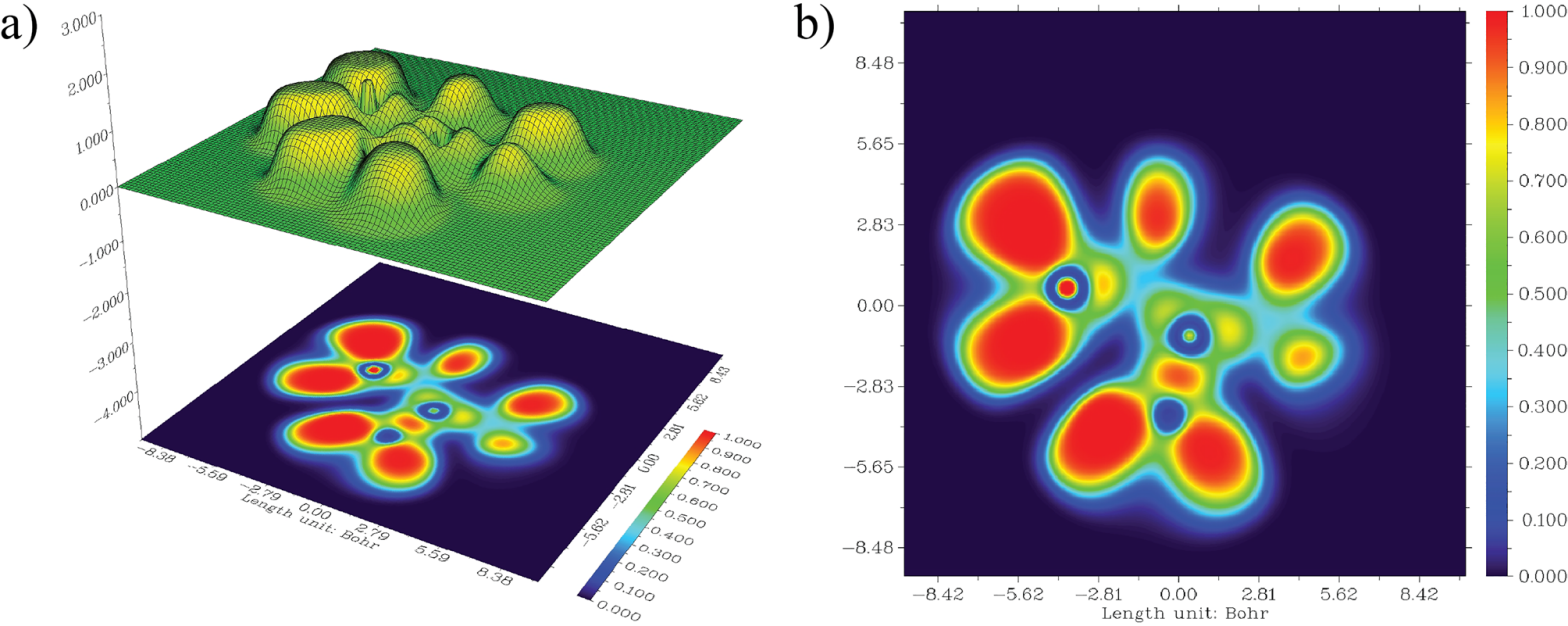
ELF plot of Neutral Withaferin A. **a** Projection of the shaded ELF surface and **b** corresponding colored ELF map.

**Fig. 6:**
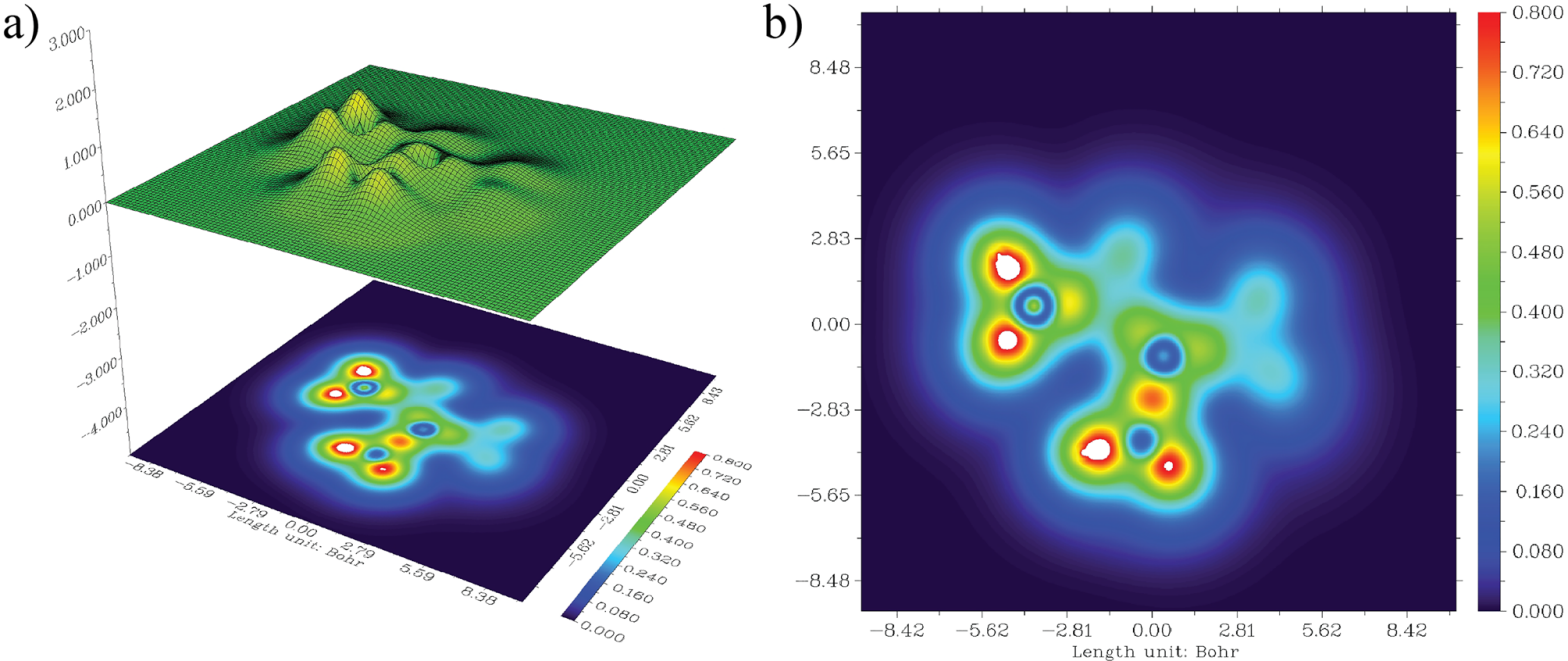
LOL plot of Neutral Withaferin A. **a** Projection of the shaded LOL surface and **b** corresponding colored LOL map.

ELF and LOL are visual descriptors that provide electron pair localization in visual representation^46^. ELF represents the probability density of finding an electron pair in a given region, whereas the LOL highlights regions of maximal orbital localization based on the gradient of the orbitals^31,43,45^. The color gradients in ELF and LOL maps illustrate the availability of non-bonding and bonding localized electrons. The red color around the hydrogen atoms shows high values of ELF, and the high localization of electrons in the presence of covalent bonds shows LOL^45^. The ELF plot in Fig. 5 with red and orange hues, typically between 0.8 and 1.0, denotes the availability of non-bonding and bonding localized electrons around chemically relevant regions near hydrogen atoms. The delocalized region, ranging from 0.0 to 0.3 and highlighted by blue circles around carbon nuclei, indicates electron-deficient areas between the valence and inner shells. The high values of the LOL, typically between 0.8 and 1.0, as indicated by the red color in the Fig. 6, are possibly due to localized lone pairs on electronegative atoms as oxygen, that indicate electron-rich zones that can be sites of reactivity or hydrogen bonding. The intermediate localization region, between 0.3 and 0.7, corresponds to the $\pi$ bonding region, and the regions with partial electron delocalization, such as conjugated systems or aromatic rings. The blue region with very low values, between 0.0 and 0.3, indicates an anti-bonding or non-bonding region between atoms.

### NCI analysis

**Fig. 7:**
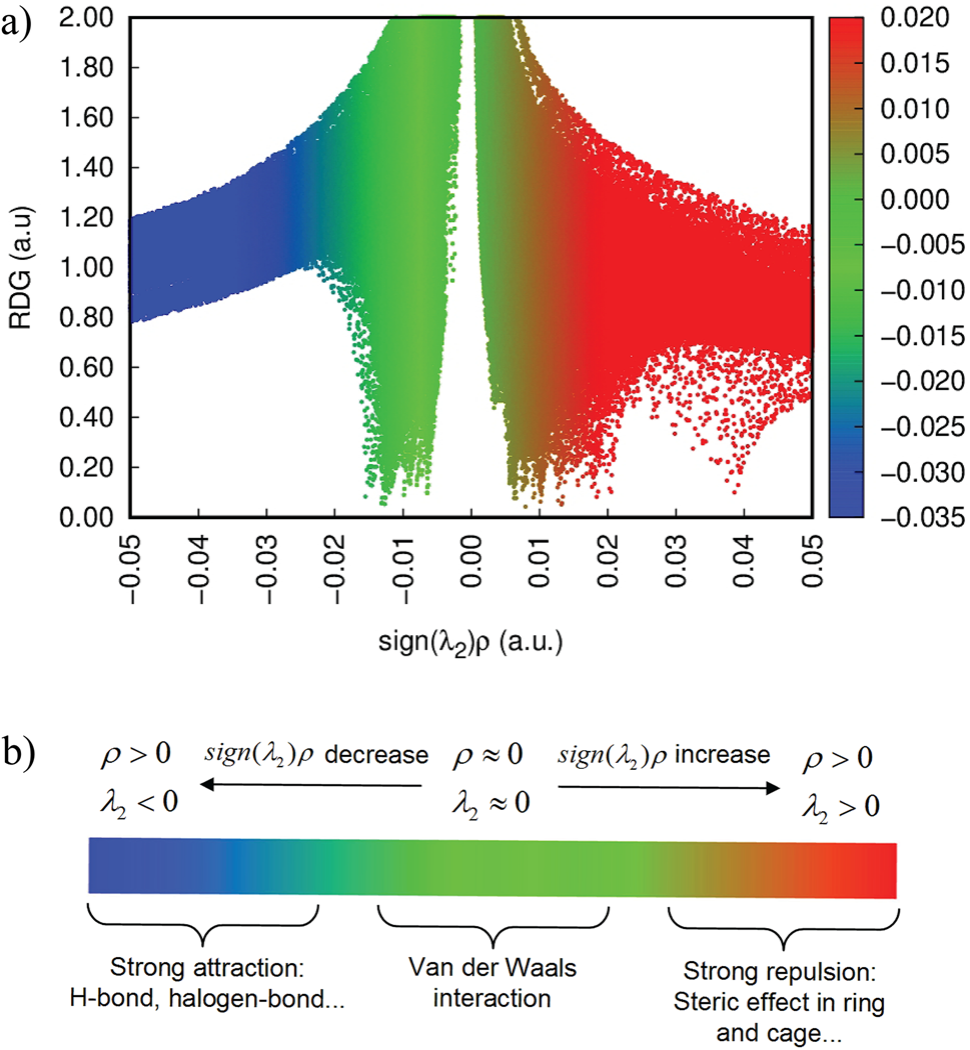
RDG scatter plot of Withaferin A in the neutral state. **a** colored RDG isosurface plot, and **b** corresponding RDG color scale bar.

Non-covalent interaction (NCI) analysis is widely employed to identify weak forces such as van der Waals interactions, steric repulsions, and hydrogen bonding^47^ . These interactions play a crucial role in determining the molecular structural stability. To probe such weak intra- and intermolecular forces, the reduced density gradient (RDG) approach was applied within the framework of NCI analysis^48^. RDG utilizes electron density and its derivatives to characterize interactions, providing a visual map of stabilizing and destabilizing effects^49^ . The RDG versus $\text {sign}(\lambda _{2})\rho$ plot Fig. 7 serves as a key indicator of interaction intensity. Here, $\lambda _{2}$ corresponds to the second-largest eigenvalue of the Hessian matrix of electron density, and its sign in combination with $\rho$ distinguishes between attractive and repulsive forces^49^. Specifically, negative $\text {sign}(\lambda _{2})\rho$ values ($< -0.02$ a.u.) represent strong attractive interactions, typically hydrogen bonding or halogen bonding, visualized as blue regions in the plot. The green regions ($-0.01$ to 0.01 a.u.) correspond to weak van der Waals interactions, which nonetheless contribute to the overall stabilization of the molecular framework. In contrast, positive $\text {sign}(\lambda _{2})\rho$ values (0.02–0.05 a.u.), shown in red, indicate steric repulsion, where atoms experience destabilizing interactions that oppose close packing.This analysis provides a comprehensive picture of how stabilizing (attractive) and destabilizing (repulsive) forces balance within the molecule, confirming the importance of weak non-covalent interactions in maintaining its structural integrity.

### Molecular Electrostatic Potential (MEP) analysis

**Fig. 8:**
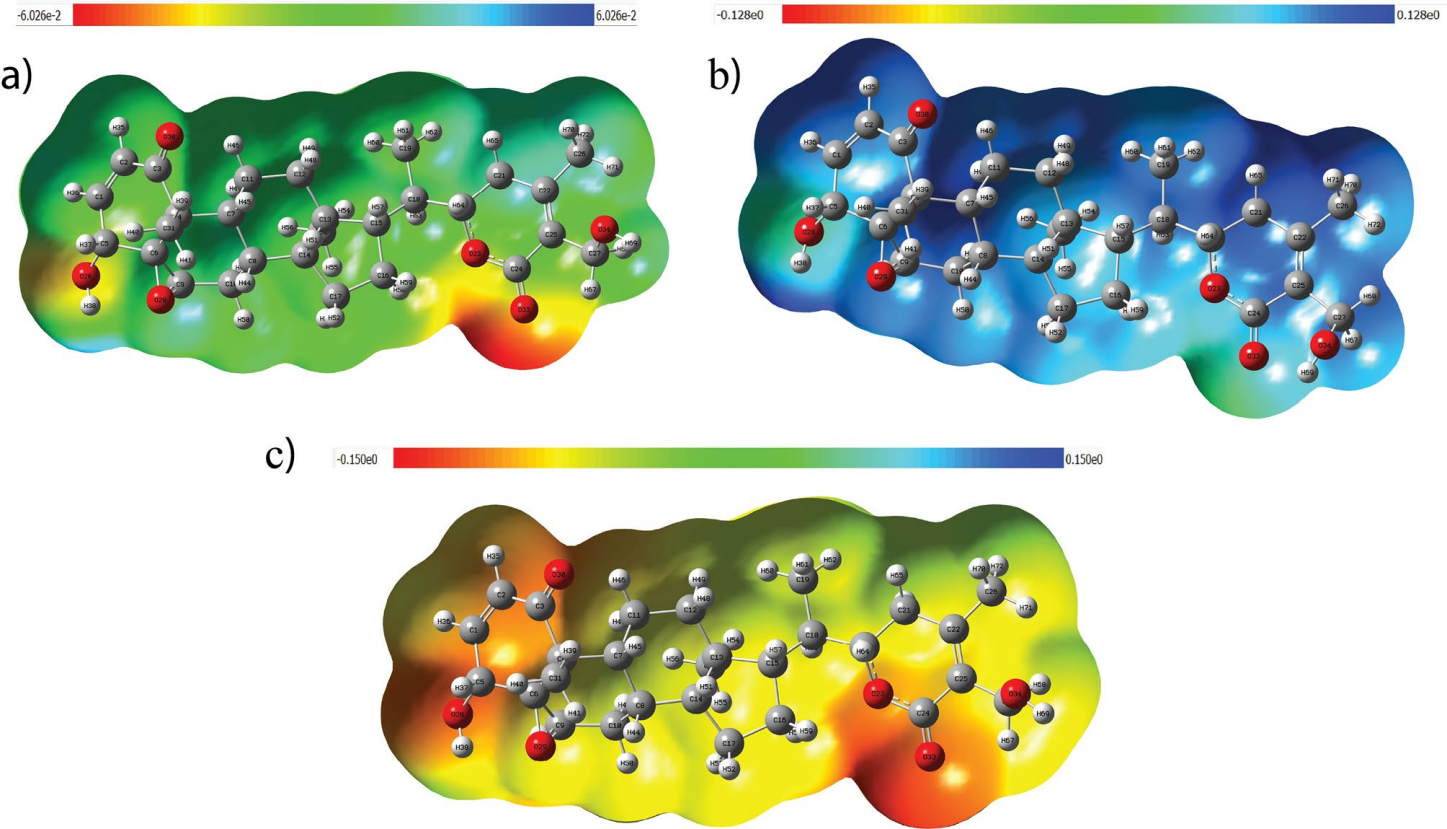
MEP of Withaferin A in different charge states: **a** Neutral, **b** Cation, and **c** Anion states.

**Fig. 9:**
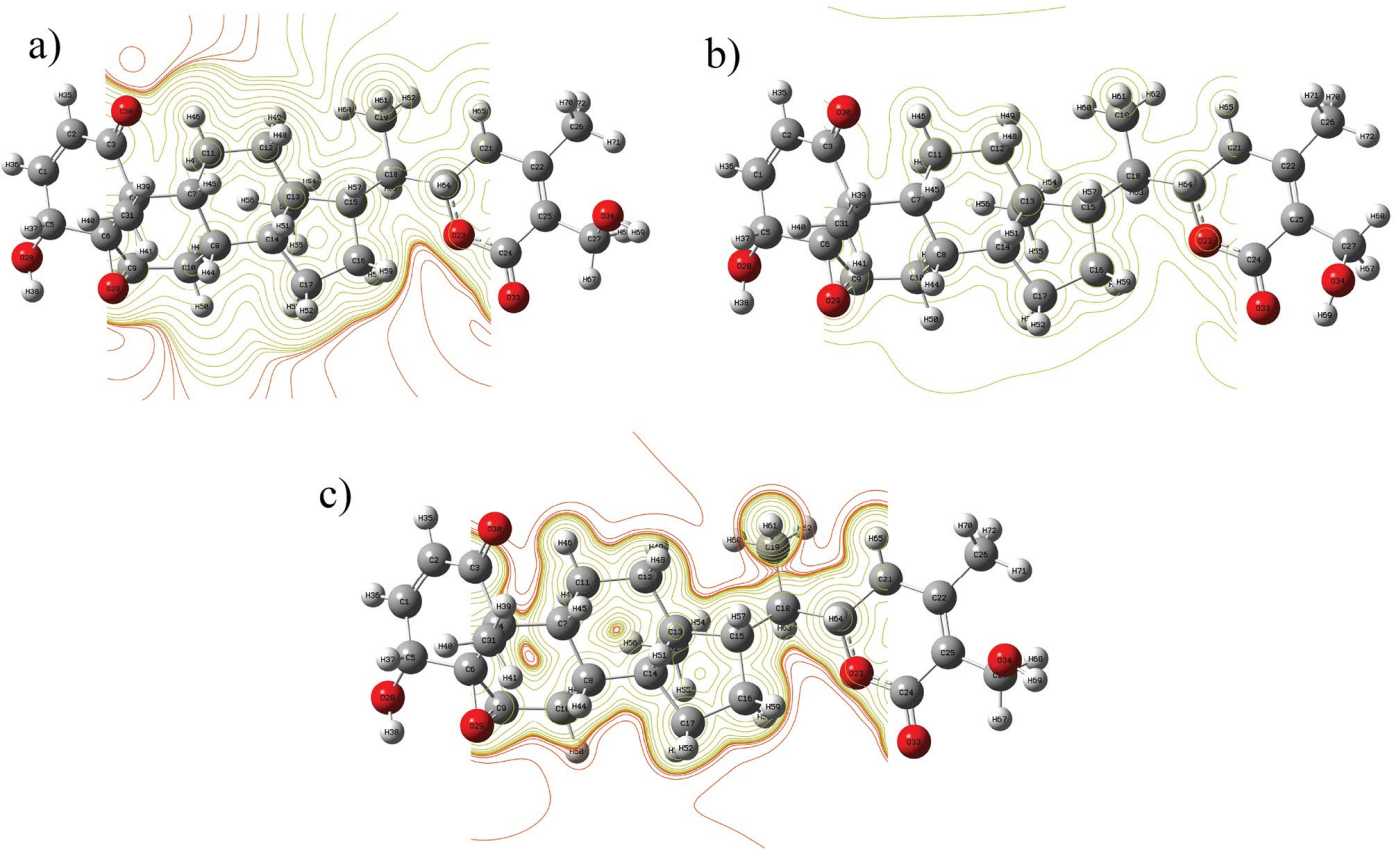
Contour plots of Withaferin A in different charge states: **a** Neutral, **b** Cation, and **c** Anion states.

Molecular electrostatic potential (MEP) mapping provides a visual representation of the electrostatic potential distribution across a molecule’s surface at constant electron density. It is a widely applied tool to predict regions susceptible to electrophilic and nucleophilic reactivity, as well as to understand intermolecular bonding interactions^31,32,42^. The method relies on electron density values at different molecular sites, with the potential surface commonly color-coded: red denotes regions of negative potential, blue indicates positive potential, and green represents neutral regions^50^. The MEP maps of the neutral, cation, and anion states shown in Fig.8a–c, respectively illustrate the charge distribution and reactive sites of the molecule. In the neutral state, high electron density localized around oxygen atoms (red regions) makes them favorable for electrophilic attacks. Upon cation formation, electron depletion around oxygen and increased potential near hydrogen and carbon atoms (blue regions) indicate enhanced susceptibility to nucleophilic attack. Conversely, in the anion state overall electron enrichment leads to reduced electrostatic potential on oxygen atoms, while comparatively positive regions (mainly hydrogen or carbons) become preferred sites for nucleophilic attack. The corresponding contour maps shown in Fig.9a–c, respectively support these findings, showing denser contours near oxygen in the neutral and anion states and sparser ones in the cation, consistent with electron gain or loss.

### Mulliken atomic charge distribution

**Fig. 10:**
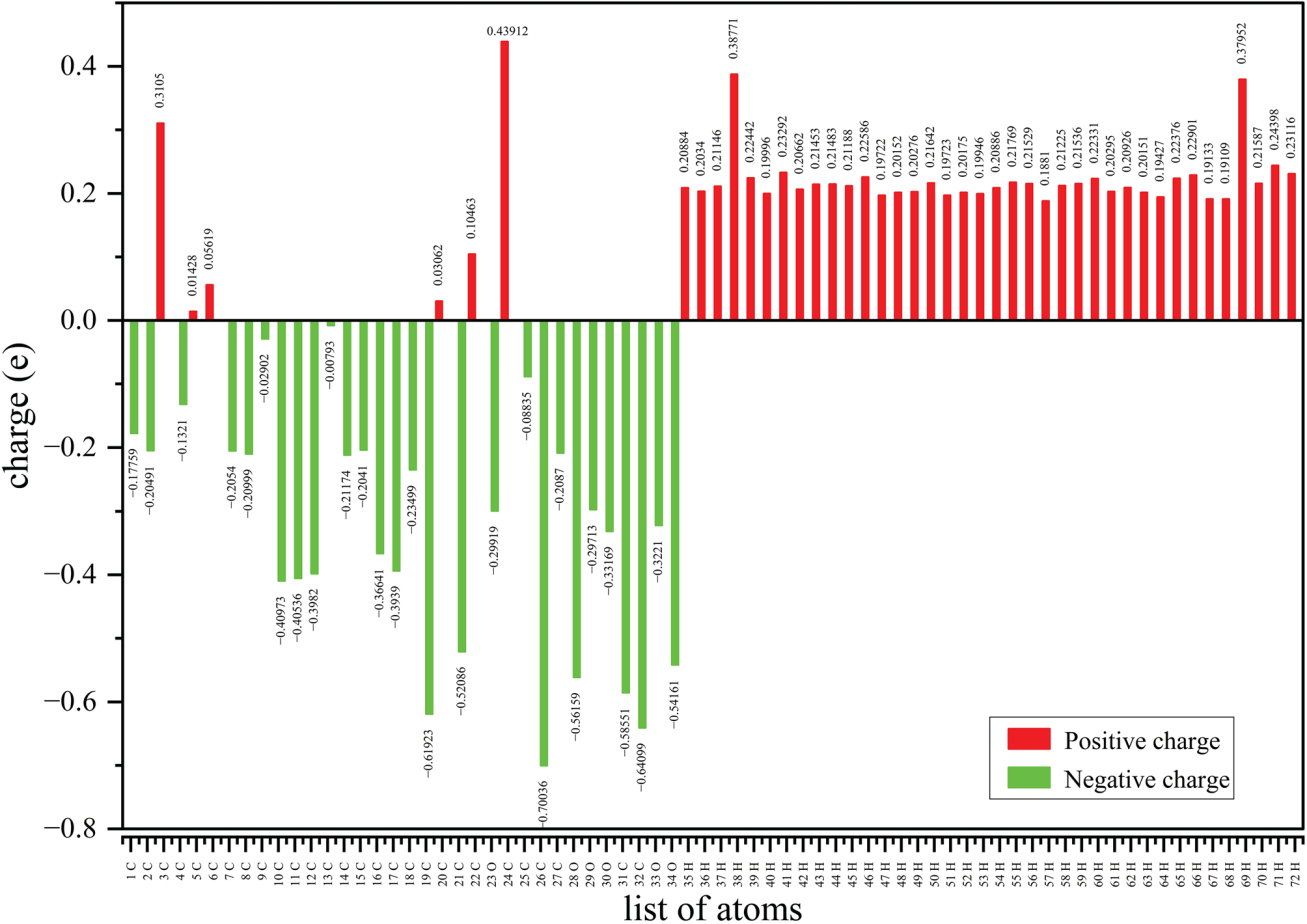
Mulliken atomic charge distribution of Withaferin A in the neutral state.

Mulliken atomic charge analysis is widely applied to determine the partial distribution of electronic charges within a molecule^35^. In quantum chemical computations, Mulliken charges play a critical role because they influence electronic structure, molecular polarizability, dipole moment, and other key physicochemical properties^47,51^. Mulliken charge distribution (Fig. 10) further supports the MEP analysis. Most hydrogen atoms carry positive charges due to electron withdrawal by nearby electronegative atoms, while carbon atoms exhibit negative charges, indicating electron richness. This asymmetric charge distribution highlights potential reactive centers; positively charged regions favor nucleophilic attack, and negatively charged ones favor electrophilic interaction, thereby providing insight into molecular reactivity and stability.

### Frontier molecular orbital (FMO) analysis

**Fig. 11:**
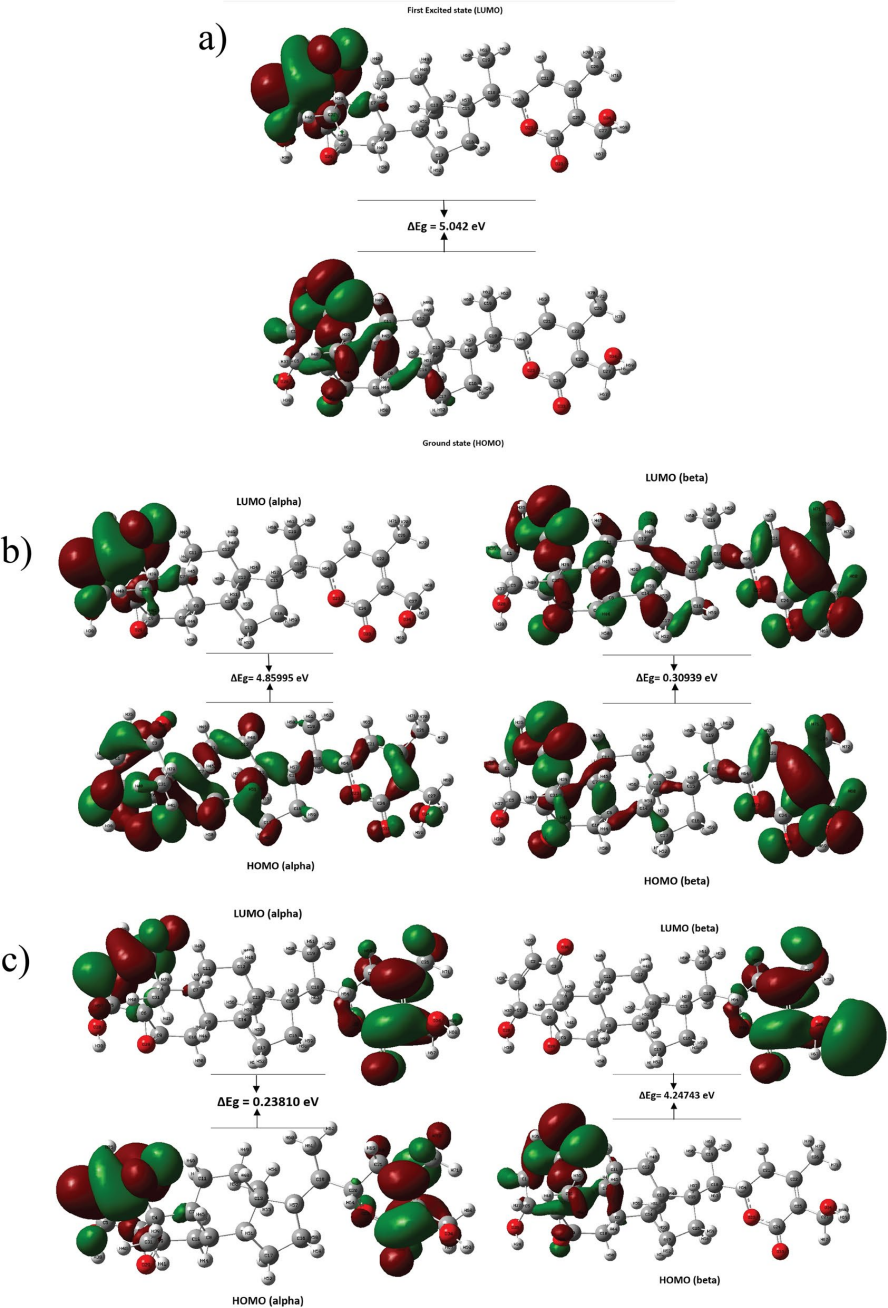
HOMO LUMO energy gap (in eV) of Withaferin A in different charge states: **a** neutral, **b** cation, and **c** anion states. The energy gap is calculated as the difference between the HOMO and the LUMO in each state, providing insights into the molecule’s electronic properties in each state.

The highest occupied molecular orbital (HOMO) and the lowest unoccupied molecular orbital (LUMO) of a molecule are collectively referred to as the frontier orbitals^32,39^. These orbitals are crucial descriptors of molecular reactivity and kinetic stability^32,39^. The LUMO represents the electron-accepting orbital, with its energy associated with the electron affinity (EA), while the HOMO represents the electron-donating orbital, with its energy related to the ionization potential^34,35,39,50^. The difference between the HOMO and LUMO energies, known as the HOMO–LUMO energy gap, provides insight into intramolecular charge transfer interactions and is a key factor in determining a molecule’s electrical transport properties^35,39^. A larger HOMO–LUMO gap generally corresponds to greater molecular stability and lower reactivity, as more energy is required to promote an electron from the HOMO to the LUMO^37^. Conversely, molecules with smaller HOMO–LUMO gaps are more reactive and less stable^35^. The frontier molecular orbitals of Withaferin A in its neutral, cation, and anion states are summarized in Table 1 and illustrated in Figs. 11a–c . The neutral state has the largest HOMO–LUMO gap (5.04 eV), indicating high kinetic stability, low chemical reactivity, and chemical hardness. The cation states show intermediate gaps, with the $\alpha$ cation (4.86 eV) being moderately stable and the $\beta$ cation (0.31 eV) being less stable and highly reactive. The anion states show varied behavior; the $\alpha$ anion (0.24 eV) has the smallest gap, indicating low stability and high chemical reactivity, while the $\beta$ anion (4.25 eV) exhibits moderate stability and reactivity.

**Table 1:** FMO energies and energy gaps of Withaferin A in different charge states. The table includes the energies of the HOMO, LUMO, and the corresponding HOMO LUMO energy gap (in eV) for the neutral, cation, and anion states.

Parameters	States				
	Neutral	$\upalpha$ Cation	$\upbeta$ Cation	$\upalpha$ Anion	$\upbeta$ Anion
ELUMO (eV)	-1.84	-5.29	-9.25	0.42	1.05
EHOMO (eV)	-6.88	-10.15	-9.56	0.18	-3.2
Energy gap = ELUMO-EHOMO = $\Delta$Eg (eV)	5.04	4.86	0.31	0.24	4.25
Stability/Reactivity	High stability, Low reactivity	Moderate stability, Moderate rectivity	Low Stability, More reactivity	Low statbility, More reactivity	Moderate stability, Moderate reactivity

### Global reactivity descriptors

Within the density functional theory (DFT) framework, commonly used global reactivity descriptors include chemical hardness ($\eta$), softness (*S*), electronegativity ($\chi$), and the electrophilicity index ($\omega$)^45,47,52^. The stability of a molecule is closely related to its hardness and softness, and these parameters provide insight into site-specific reactivity and potential hazardous behavior^47^. The ionization potential (*I*) represents the energy required to remove an electron and form a positively charged ion^53^, whereas the electron affinity (*EA*) corresponds to the energy released upon the addition of an electron to a neutral atom or molecule, forming a negatively charged ion^54^. Electronegativity ($\chi$) quantifies an atom’s ability to attract electrons in a covalent bond^55^, and the chemical potential ($\mu$) describes the tendency of electrons to escape from the molecular system, with higher $\mu$ values indicating increased reactivity and lower stability^56^. Chemical hardness ($\eta$) reflects resistance to electron density redistribution, and softness (*S*), the reciprocal of hardness ($S = 1/\eta$), measures the ease of electronic deformation^31,45,56^. The electrophilicity index ($\omega$) serves as a descriptor of a molecule’s electron-accepting capability, where higher $\omega$ values indicate strong electrophilic behavior and lower values suggest nucleophilic character^47,57^ . In our study, the $\beta$ cation exhibits notably high electrophilicity ($\omega = 285.91$ eV) and softness ($S = 30.90$ $\hbox {eV}^{-1}$), indicating pronounced electron-accepting behavior and elevated chemical reactivity. In contrast, the $\alpha$ anion displays a negative softness value, suggesting computational instability or potential methodological limitations. The chemical potential decreases (becomes more negative) from the neutral to the cation state, reflecting increasing electron deficiency, whereas the transition from neutral to anion states reduces electron deficiency, consistent with nucleophilic behavior. These variations demonstrate charge-dependent reactivity, with cation species acting predominantly as electrophiles and anion species as nucleophiles. Detailed numerical values of global reactivity descriptors for all charge states are provided in Table 2.

**Table 2:** Calculated global reactivity descriptors of Withaferin A in different charge states: neutral, $\upalpha$-cation, $\upbeta$-cation, $\upalpha$-anion, and $\upbeta$-anion. he table includes HOMO and LUMO energies (in eV), energy gap ($\Delta$Eg) (in eV), ionization potential (I), electron affinity (A), electronegativity ($\upchi$), chemical potential ($\upmu$), chemical hardness ($\upeta$), softness (S), and electrophilicity index ($\upomega$). All calculations were performed using DFT at the B3LYP/6-311G(d) level.

Parameters	States				
	Neutral	$\upalpha$ Cation	$\upbeta$ Cation	$\upalpha$ Anion	$\upbeta$ Anion
ELUMO (eV)	-1.84	-5.29	-9.25	0.42	1.05
EHOMO (eV)	-6.88	-10.15	-9.56	0.18	-3.20
Energy gap = ELUMO-EHOMO = $\Delta$Eg (eV)	5.04	4.86	0.31	0.24	4.25
Ionization potential [I = EHOMO] (eV)^58,59^	6.88	10.15	9.56	-0.18	3.20
Electron affinity [A = ELUMO] (eV)^58,59^	1.84	5.29	9.25	-0.42	-1.05
Electronegativity [$\upchi$ = (I +A)/2] (eV)^58,59^	4.36	7.72	9.41	-0.30	1.07
Chemical potential [$\upmu$ = $\upchi$ = - (I +A)/2] (eV)^59^	-4.36	-7.72	-9.41	0.30	-1.07
Chemical hardness [$\upeta$ = (I A)/2] (eV)^58,59^	2.52	2.43	0.15	0.12	2.12
Chemical softness [S = I/2$\upeta$] (eV)-1^59^	1.37	2.09	30.90	-0.76	0.75
Electrophilicity index [$\upomega$ = $\upmu$2/2$\upeta$] (eV)^58,59^	3.77	12.26	285.91	0.38	0.27

### Density of states (DOS) analysis

**Fig. 12:**
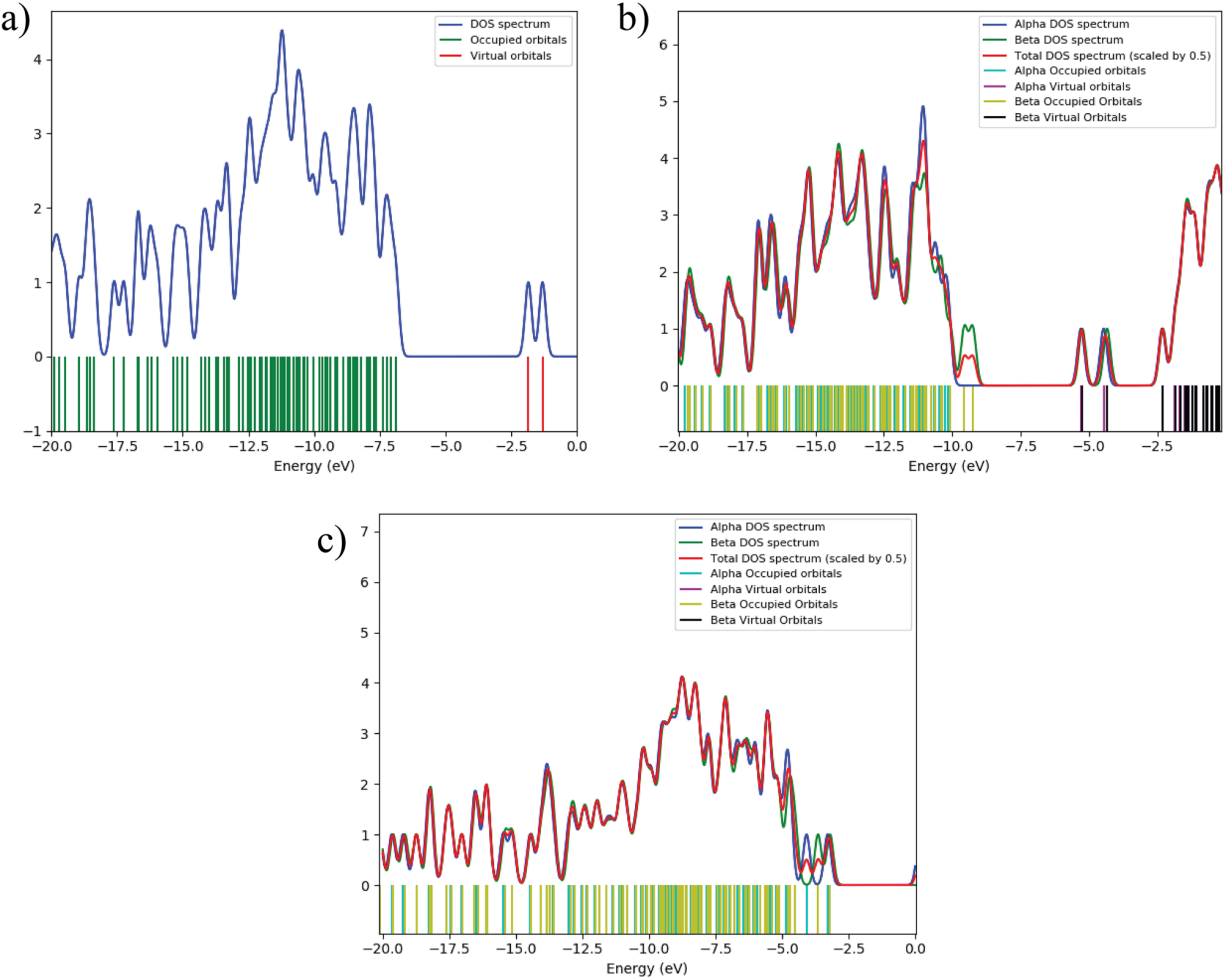
Density of States (DOS) of Withaferin A in different charge states: **a** neutral, **b** cation, and **c** anion states. The figure illustrates the electronic structure and distribution of energy levels in each charge state.

The density of states (DOS) of a molecule provides a detailed description of the distribution of energy levels per unit energy interval and their orbital contributions. DOS plots visualize the electron density associated with each molecular orbital, offering a comprehensive representation of characteristic orbitals within a defined energy range. In these plots, the HOMO and LUMO levels are typically highlighted, with red and green lines representing the respective orbitals. A pronounced DOS peak at a given energy level indicates a high density of accessible electronic states, whereas zero intensity signifies the absence of states at that energy^35^. In this study, the DOS of Withaferin A was calculated using GaussSum 3.0 software with full width at half maximum (FWHM)^23^. The DOS spectra for Withaferin A in its neutral, cation, and anion states are shown in Fig. 12a–c, respectively, covering the energy range from –25 eV to 0 eV. The color scheme of the DOS spectrum represents orbital phase, where green corresponds to the negative phase and red corresponds to the positive phase of molecular orbitals^60^. For the neutral state, filled orbitals (donor states) occupy the energy range from –20.0 eV to –7.5 eV, whereas virtual orbitals (acceptor states) are observed between –5 eV and 0 eV^35^. The energy gaps derived from HOMO–LUMO analysis are in excellent agreement with those observed in the DOS spectra, confirming the reliability of the electronic structure calculations.

### Thermodynamic properties

**Fig. 13:**
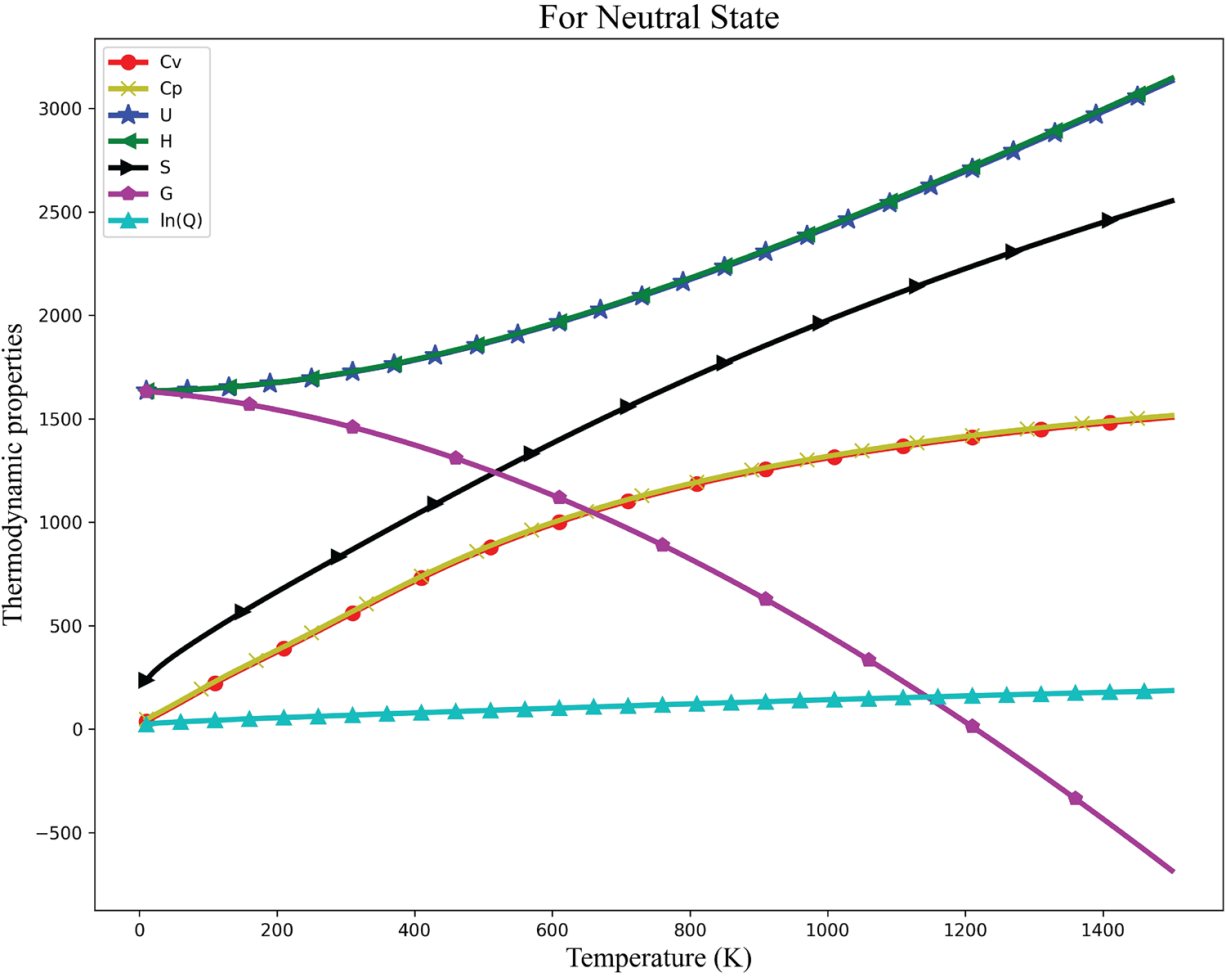
Variation of thermodynamic variables with temperature for Withaferin A. The figure shows how parameters such as thermal energy, specific heat, entropy, enthalpy, and other thermodynamic properties change as a function of temperature.

Thermodynamic properties play a vital role in understanding the kinetics, selectivity, and feasibility of chemical processes. Quantum computational approaches, such as density functional theory (DFT), provide reliable estimates of thermodynamic parameters, including enthalpy, entropy, and related quantities essential for predicting reaction behavior^61^. In this study, the thermodynamic functions of Withaferin A were derived from vibrational analysis, taking into account contributions from electronic, translational, vibrational, rotational, and zero-point vibrational energies. The complete set of thermodynamic parameters for the neutral state is summarized in Table 3. As illustrated in Fig. 13, increasing temperature leads to a rise in heat capacities ($C_v$, $C_p$), internal energy (*U*), enthalpy (*H*), entropy (*S*), and the partition function ($\ln Q$), while the Gibbs free energy (*G*) decreases. This behavior indicates enhanced molecular disorder and increased spontaneity of processes at higher temperatures. The temperature dependence of these thermodynamic properties demonstrates the variations of key molecular parameters over the studied temperature range, providing insight into the thermal stability and reactivity of the molecule.

**Table 3:** Thermodynamic parameters at 298.15 K for Withaferin A, calculated at the B3LYP/6-311G(d) level. The table includes contributions from electronic, translational, rotational, and vibrational components for thermal energy (in kcal/mol), specific heat (in cal/mol$\cdot$K), and entropy (in cal/mol$\cdot$K). Zero-point vibrational energy (ZPVE) is also reported.

Parameters	Thermal Energy	Specific Heat	Entropy
	(KCal/Mol)	(Cal/Mol-Kelvin)	(Cal/Mol-Kelvin)
Electronic	0	0	0
Translational	0.889	2.981	44.333
Rotational	0.889	2.981	37.39
Vibrational	409.064	123.273	120.8
Total	410.841	129.235	202.523
Zero-Point Vibrational Energy	390.56233	*	*

### Fukui function analysis

The Fukui function is a key reactivity descriptor used to identify regions of a molecule most susceptible to electrophilic or nucleophilic attacks under a constant external potential^31,62^. The functions $f_j^{+}$ and $f_j^{-}$ correspond to nucleophilic and electrophilic attack indices, respectively, reflecting the tendency of an atomic site to gain or lose electrons^31,42,63^. The condensed Fukui functions for the *k*th atomic site are calculated as follows^62^:

2 $$\begin{aligned} \begin{aligned} f^+_j = q_j(N+1) - q_j(N) \\ f^-_j = q_j(N) - q_j(N-1) \\ f^0_j = \frac{1}{2}\left[ q_j(N+1) - q_j(N-1)\right] \end{aligned} \end{aligned}$$

where $q_j$ denotes the atomic charge at site *j*^4,34,42,43^. The dual descriptor $\Delta f(r)$, defined as^43,62^:

3 $$\begin{aligned} \Delta f(r) = f^+_k - f^-_k \end{aligned}$$

helps to identify reactive regions: regions with $\Delta f(r) > 0$ are favorable for nucleophilic attack, while $\Delta f(r) < 0$ indicates a preference for electrophilic attack^43,62^.

The Fukui function analysis, performed using the UCA-FUKUI software^62^, quantitatively identifies specific reactive sites within the molecule. Atoms C1 and C3, with high $f^{+}$ values, exhibit pronounced nucleophilic character, whereas O34, with an elevated $f^{-}$ value, serves as an electrophilic center. The O30 atom displays contributions from both $f^{+}$ and $f^{-}$, suggesting resonance stabilization, while hydrogen atoms exhibit negative $\Delta f(r)$ values, confirming their electrophilic nature. These observations are consistent with the molecular electrostatic potential (MEP) and Mulliken population analyses, collectively validating the predicted charge delocalization and chemical reactivity patterns. Detailed numerical values of the Fukui function for all atoms are summarized in Supplementary Table S8.

### Molecular docking analysis

Molecular docking is a powerful computational approach widely used to study biomolecular interactions in drug design and discovery^45,49^. This technique enables the prediction of both binding orientations and interaction types between ligands and protein active sites^45,64^.

**Fig. 14:**
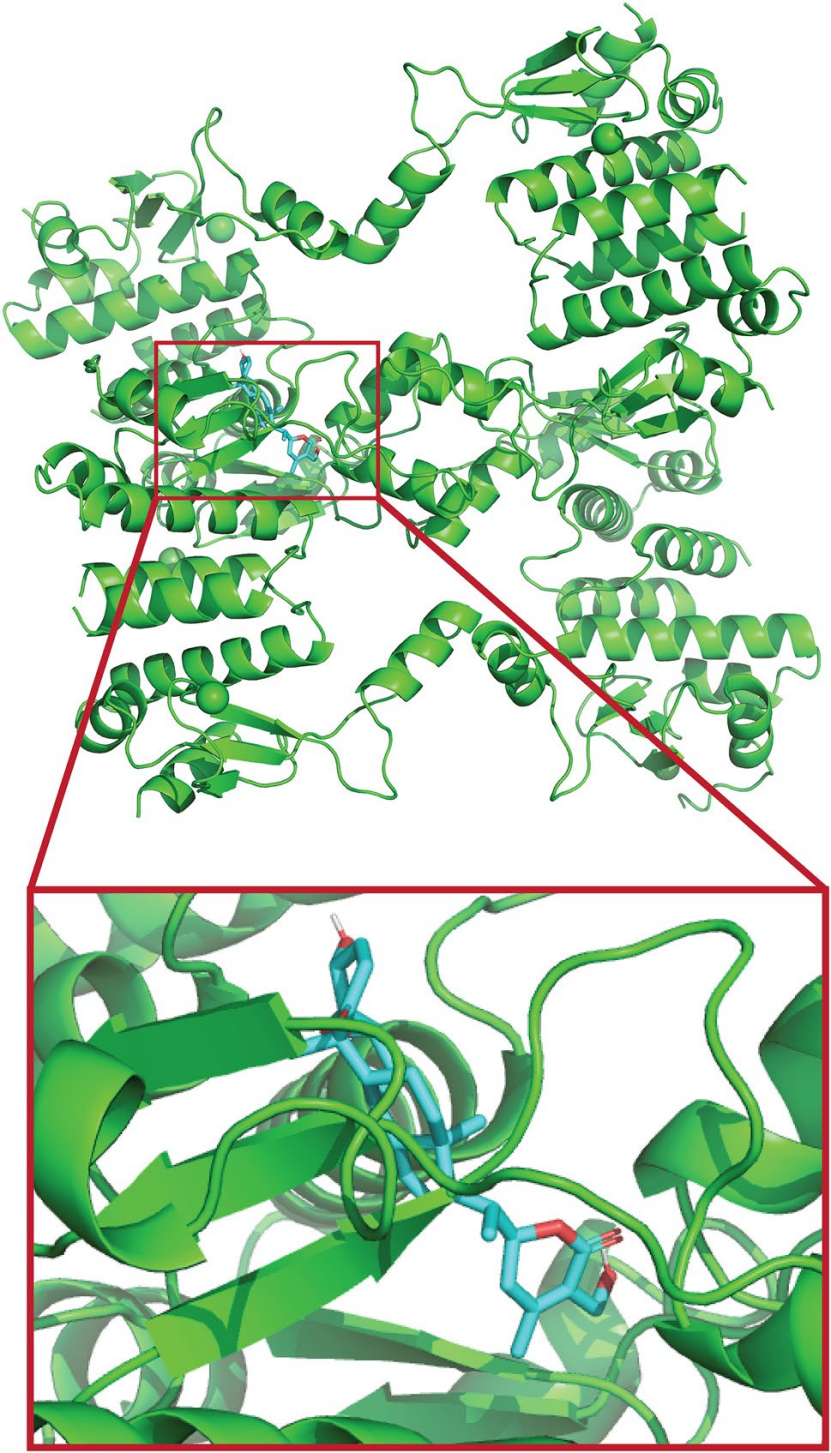
Interaction of Withaferin A with Hsp90: 3D plot showing the binding interaction.

In the present study, Withaferin A was docked within the binding cavity of the target protein Hsp90 to determine its preferred binding orientation and binding affinity, as illustrated in Fig. 14. The structure of Hsp90 (PDB ID: 1SF8 | pdb_00001sf8) was obtained from the Protein Data Bank and prepared using AutoDock Vina (v1.5.7). During protein preparation, all crystallographic water molecules were removed to prevent interference with ligand binding, as bounded water molecules within the binding pocket may hinder the optimal ligand accommodation. Polar hydrogens were subsequently added, and Kollman charges were assigned to ensure accurate electrostatic representation. Withaferin A (neutral form) was prepared as the ligand and converted to PDBQT format in AutoDockTools using default settings optimized for AutoDock Vina. Docking calculations were performed using a grid box centered at (52.718, 10.805, 1.736) Å with dimensions of 40 $\times$ 40 $\times$ 40 Å along the *x*, *y*, and *z* axes, respectively. The energy range was set to 4 kcal $\hbox {mol}^{-1}$, and an exhaustiveness value of 8 was used to balance computational efficiency with accuracy. The default Vina scoring function was employed to rank the poses, and nine binding modes were generated for the ligand.

**Fig. 15:**
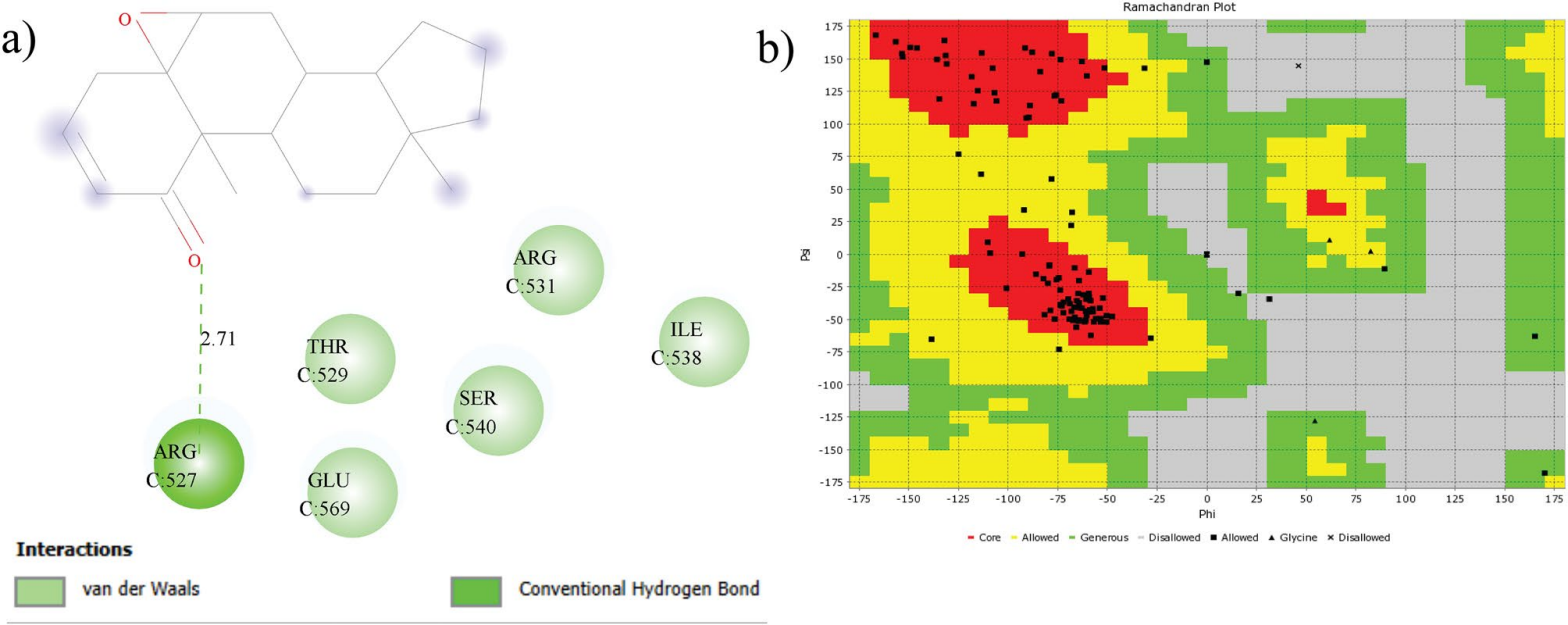
**a** 2D plot highlighting the key interaction sites and residues involved in the binding, **b** Ramachandran plot of Withaferin A.

Docking of Withaferin A into the Hsp90 binding cavity produced nine distinct binding modes with predicted affinities ranging from –9.3 to –8.2 kcal $\hbox {mol}^{-1}$ (Table 4), indicating a favorable interaction with the target. The best-scoring pose (-9.3 kcal $\hbox {mol}^{-1}$) was selected for further analysis and used as the reference for RMSD calculations. As illustrateed in (Fig. 15a), ARG C:527 formed a conventional hydrogen bond with the ligand, while THR C:529, GLU C:569, ARG C:531, SER C:540, and ILE C:538 participated in van der Waals interactions, collectively stabilizing the ligand-protein complex. Validation by redocking geldanamycin yielded a predicted affinity of –7.9 kcal $\hbox {mol}^{-1}$, consistent with previous reports in which geldanamycin and its derivatives were docked, and 17-DMAG exhibited the highest binding affinity (–7.73 ± 0.12 kcal $\hbox {mol}^{-1}$)^65^, thereby supporting the reliability of the docking protocol. The Ramachandran plot (Fig. 15b) confirmed the stability of the target protein, with over 90% of amino acid residues located in favored regions and minimal residues in disallowed regions, suggesting an overall stable protein conformation suitable for docking studies.

These results indicate strong and consistent ligand-protein interactions, optimal binding site geometry, and potential for effective biological activity. Collectively, these computational findings highlight the potential of Withaferin A as a promising ligand for Hsp90 inhibition and provide a strong basis for further experimental validation.

**Table 4:** Docking modes of Withaferin A (ligand) with the Hsp90 protein, ranked by binding affinity (kcal/mol). RMSD l.b. and RMSD u.b. represent the lower and upper bounds of the root-mean-square deviation (RMSD) with respect to the best binding mode. Mode 1 represents the best pose with the highest binding affinity (-9.3 kcal/mol).

Mode	Affinity	dist. from best mode	
	(kcal/mol)	RMSD l.b.	RMSD u.b.
1	-9.3	0	0
2	-8.7	3.289	5.445
3	-8.6	1.301	2.658
4	-8.5	6.427	13.945
5	-8.5	1.757	9.538
6	-8.3	3.635	8.321
7	-8.3	1.946	8.884
8	-8.3	3.51	8.926
9	-8.2	5.902	11.353

## Conclusion

This study provides a comprehensive computational insight into the structural stability, electronic reactivity, and biological potential of Withaferin A. The analyses indicate extensive electron delocalization, significant hyperconjugative interactions, and balanced electrophilic nucleophilic character, confirming its structural robustness and high kinetic stability. Reactive regions identified through MEP and NCI analyses, along with favorable charge-transfer properties from frontier molecular orbitals, further underscore its electronic versatility. Thermodynamic results demonstrate favorable stability and spontaneity across varying conditions, supporting potential chemical and biological activity.

Molecular docking with Hsp90 revealed strong ligand protein binding, optimal orientation, and stabilizing interactions, highlighting Withaferin A as a potent and stable inhibitor with higher affinity than some standard inhibitors.

Overall, Withaferin A emerges as a structurally stable, electronically versatile, and biologically relevant molecule with promising therapeutic potential. These computational findings provide a strong molecular-level framework to guide future experimental validation, structure activity relationship studies, and the rational design of potent Hsp90-targeted inhibitors.

## Supplementary Information

Supplementary Information.

## Data Availability

The datasets generated and analyzed during the current study are available from the corresponding author on reasonable request. The data supporting this article have been included as part of the supplementary information.
