# Supplementary material for: DFT supported computational insights into Withaferin A structure and its binding interaction with Hsp90
Source: Sci Rep. 2025 Dec 29;16:3235. doi: 10.1038/s41598-025-33145-w (PMC12830739; doi:10.1038/s41598-025-33145-w)
Supplement: Supplementary file 1 — Supplementary Information. [file 41598_2025_33145_MOESM1_ESM.pdf]

Supplementary Information: DFT Supported Computational Insights into Withaferin A Structure and Its Binding Interaction with Hsp90

Romash Shoni[1], Phadindra Raj Karki[1], and Khagendra Tripathi[1,*]

[1]Department of Physics, St. Xavier's College, Tribhuvan University, Kathmandu, Nepal
[*]Corresponding author: tripathikhagendra25@gmail.com / ktripathi@sxc.edu.np

Supplementary Tables

Table S1: PED Analysis–Neutral State: This table presents the Potential Energy Distribution (PED) of vibrational modes for the neutral state of Withaferin A, computed at the B3LYP/6-311G(d) level. Mode assignments and dominant internal coordinate contributions are included. Modes without assignments have low or distributed contributions, indicating mixed, delocalized, or weak vibrational motions. Here, ν denotes stretching vibrations, β denotes bending vibrations, and δ denotes torsional vibrations. Vibrational modes with less than 10% PED contribution aren't assigned.

Frequencies calculated (cm^{-1})		IR intensity	Raman Intensity	Assignment
Unscaled	Scaled[a]			
3780.13	3656.1417	8.4229	248.7551	ν OH (100)
3754.81	3631.6522	23.5551	65.2338	ν OH (100)
3191.14	3086.4706	11.7596	204.3749	ν CH (97)
3170.86	3066.8558	2.5874	86.8271	ν CH (97)
3158.83	3055.2204	13.4821	79.8934	ν CH (85) + ν CH (12)
3148.56	3045.2872	31.8317	84.058	ν CH (87)
3142.88	3039.7935	8.76	43.2022	ν CH (99)
3139.4	3036.4277	30.4177	108.2705	ν CH (88)
3132.81	3030.0538	34.4897	54.9946	ν CH (87)
3119.87	3017.5383	36.461	44.3462	ν CH (87)
3112.29	3010.2069	2.6826	9.8504	ν CH (80)
3111.34	3009.2880	29.4642	47.4094	ν CH (91)
3107.41	3005.4870	17.0893	69.1642	ν CH (82)
3104.05	3002.2372	57.066	124.1206	ν CH (80)
3101.23	2999.5097	37.7852	57.8139	ν CH (86)
3085.64	2984.4310	15.3669	43.2469	ν CH (97)
3082.55	2981.4424	48.2471	119.2565	ν CH (85)

Table S1 continued from previous page

3081.15	2980.0883	22.2138	93.299	ν CH (75) + ν CH (12)
3073.17	2972.3700	55.1558	66.1962	ν CH (67) + ν CH (13)
3068.43	2967.7855	17.0866	62.1116	ν CH (85)
3061.51	2961.0925	14.5816	81.1888	ν CH (96)
3054.55	2954.3608	23.8619	141.1158	ν CH (91)
3046.42	2946.4974	38.3988	177.6978	ν CH (95)
3044.17	2944.3212	22.1463	106.9541	ν CH (78)
3043.39	2943.5668	67.1134	30.1849	ν CH (77)
3039.07	2939.3885	41.6492	6.2271	ν CH (82)
3037	2937.3864	6.9588	21.0515	ν CH (82)
3033.82	2934.3107	8.0786	70.8483	ν CH (12) + ν CH (75)
3030.28	2930.8868	13.5339	51.1122	ν CH (83)
3022.44	2923.3040	25.3794	200.9256	ν CH (11) + ν CH (82)
3020.71	2921.6307	2.9201	36.4398	ν CH (83)
3014.11	2915.2472	23.4765	59.3813	ν CH (14) + ν CH (77)
3011.16	2912.3940	23.4282	15.6168	ν CH (11) + ν CH (77)
2998.93	2900.5651	6.3491	120.881	ν CH (76)
2987.57	2889.5777	16.7342	22.1081	ν CH (73) + ν CH (12)
2974.28	2876.7236	71.6868	141.6997	ν CH (97)
2968.09	2870.7366	5.995	9.8958	ν CH (12) + ν CH (81)
2952.29	2855.4549	32.2331	94.8044	ν CH (99)
1784.19	1725.6686	385.3096	38.1349	ν CC (81)
1743.99	1686.7871	175.4029	34.9942	ν CC (72)
1705.83	1649.8788	33.0916	40.4974	ν CC (25) + ν CC (10)
1683.73	1628.5037	6.2626	38.1285	ν CC (30)
1546.48	1495.7555	8.5795	9.9208	β HCO (58)
1542.61	1492.0124	7.3571	8.6307	β HCH (30) + β HCC (14)
1539.96	1489.4493	10.6425	7.1805	δ HCCC (12)
1532.68	1482.4081	0.8328	2.6804	
1528.51	1478.3749	3.4423	4.3612	δ HCCC (22)
1523.42	1473.4518	5.6932	7.0894	δ HCCC (30)
1521.69	1471.7786	6.658	4.1591	δ HCCC (24)
1512.51	1462.8997	1.7085	2.2737	δ HCCC (14)
1510.95	1461.3908	7.0127	7.0631	δ HCCC (36)
1503.26	1453.9531	11.8584	1.4855	δ HCCC (14) + β HCO (12)
1502.08	1452.8118	6.3837	13.307	β HCC (10) + β HCO (10) + δ HCCC (23)
1500.07	1450.8677	0.9146	14.7917	
1497.86	1448.7302	3.7438	7.3645	
1488.99	1440.1511	8.2452	17.4658	δ HCCC (34)
1482.86	1434.2222	11.6472	5.8111	ν CC (13)
1479.9	1431.3593	5.8839	11.6514	β HCC (10)
1473.46	1425.1305	16.7487	3.0851	
1453.13	1405.4673	3.3854	3.0416	β HCC (15)
1447.64	1400.1574	3.9646	6.285	β HCC (18)
1436.56	1389.4408	9.404	1.1914	β HCC (24)
1432.54	1385.5527	9.6597	1.9328	β HCH (13) + β HCH (10)
1429.01	1382.1385	23.3999	21.0375	β HCC (33)
1420.92	1374.3138	12.2823	1.1093	β HCH (11)
1412.36	1366.0346	9.5296	0.5226	β HCH (11)
1411.67	1365.3672	20.7307	1.2609	
1409.4	1363.1717	7.0467	2.9466	
1408.85	1362.6397	5.1667	1.8996	
1400.3	1354.3702	7.4629	4.2918	
1390.69	1345.0754	3.3294	5.7067	β HCC (12)
1383.69	1338.3050	6.4574	0.9335	
1382.98	1337.6183	11.0513	10.3533	
1371.58	1326.5922	42.903	3.6821	
1364.8	1320.0346	47.734	8.3692	
1363.96	1319.2221	3.4229	3.9743	
1359.27	1314.6859	0.1146	6.5348	
1353.57	1309.1729	3.8462	0.5479	
1341.72	1297.7116	4.0394	3.0229	
1338.2	1294.3070	1.2841	4.7559	
1331.96	1288.2717	30.1126	6.5647	
1327.38	1283.8419	12.7929	2.6848	
1323.99	1280.5631	5.6989	2.1637	δ HCCC (10)
1321.63	1278.2805	38.3997	10.142	

Table S1 continued from previous page

1304.66	1261.8672	4.6823	3.9522	
1302.33	1259.6136	6.5285	7.1951	
1290.65	1248.3167	1.9297	6.3636	β HCC (12)
1269.18	1227.5509	7.8766	1.9182	β HCC (14)
1263.37	1221.9315	9.8212	4.8718	
1260.89	1219.5328	100.403	4.246	β HCC (10)
1259.71	1218.3915	41.7743	9.3005	
1252.34	1211.2632	21.5134	6.7833	
1249.71	1208.7195	41.0467	2.3749	
1246.4	1205.5181	55.2394	4.3618	
1237.8	1197.2002	2.7291	10.6785	
1227.88	1187.6055	42.5732	3.8097	
1224.23	1184.0753	7.3614	5.3842	
1203.98	1164.4895	91.9165	3.5947	ν CC (14)
1191.99	1152.8927	3.313	3.1729	
1186.81	1147.8826	6.2788	1.3185	
1168.78	1130.4440	10.4698	3.9408	
1165.29	1127.0685	20.1748	1.2627	
1161.04	1122.9579	12.2727	2.9888	
1151.42	1113.6534	63.5049	8.6353	
1139.12	1101.7569	22.6921	3.8891	
1136.99	1099.6967	61.5725	2.0508	ν CC (12)
1128.41	1091.3982	35.2664	1.5856	ν CC (11)
1115.66	1079.0664	7.3343	3.0088	
1108.91	1072.5378	9.3904	1.3027	
1099.51	1063.4461	32.0469	3.7419	
1096.97	1060.9894	48.6096	5.2142	
1094.9	1058.9873	38.5297	2.6076	
1087.29	1051.6269	4.1629	4.5504	
1079.25	1043.8506	3.3586	3.5644	ν CC (22)
1072.02	1036.8577	7.0769	1.7655	
1064.3	1029.3910	7.5291	3.7203	
1056.51	1021.8565	10.4695	4.4982	
1050.49	1016.0339	6.5647	2.2451	
1044.73	1010.4629	84.7649	3.0671	
1033.11	999.2240	13.9011	3.4451	ν CC (24)
1025.13	991.5057	15.5226	1.9546	
1021.07	987.5789	8.7654	8.6839	
1018.45	985.0448	3.036	6.9574	
1011.13	977.9649	7.495	0.313	
1008.31	975.2374	6.2576	6.6468	
992.37	959.8203	8.4445	4.5799	ν CC (15)
986.99	954.6167	9.3152	1.5735	ν CC (12)
982.45	950.2256	3.1236	5.0463	
971.26	939.4027	6.1787	1.1221	ν CC (12)
968.51	936.7429	12.0857	7.6139	
956.21	924.8463	13.9391	4.3793	
953.03	921.7706	5.8974	4.2822	
942.26	911.3539	14.1681	1.7693	
935.91	905.2122	3.6237	3.2667	
916.44	886.3808	4.8801	1.1524	
910.47	880.6066	11.0432	4.0708	
881.68	852.7609	12.769	1.1329	
877.6	848.8147	7.646	4.2138	
858.28	830.1284	14.7931	1.0005	
853.09	825.1086	24.3562	2.5369	
843.76	816.0847	3.5823	2.7136	
807.76	781.2655	3.0708	5.9186	ν CC (16)
800.3	774.0502	3.6891	1.6102	
793.16	767.1444	14.7804	1.6363	
788.83	762.9564	4.396	3.7627	ν CC (10)
761.54	736.5615	2.5735	3.4198	
742.89	718.5232	21.5648	3.7268	
729.56	705.6304	3.3838	4.1734	ν CC (21)
722.76	699.0535	12.1331	4.4739	
709.1	685.8415	7.3965	6.9661	
674.49	652.3667	14.2999	1.0719	

Table S1 continued from previous page

652.19	630.7982	1.8106	3.3628	
636.06	615.1972	3.1646	3.9321	
623.14	602.7010	1.2191	4.0206	
611.19	591.1430	4.0241	0.7748	
601.09	581.3742	3.8791	0.9156	
578.28	559.3124	2.8928	6.7756	
553.37	535.2195	1.0766	1.2327	
552.81	534.6778	5.7202	0.6945	
541.96	524.1837	1.8401	1.8362	
537.89	520.2472	8.2978	2.9722	
510.94	494.1812	10.315	2.3521	β HOC (11)
498.85	482.4877	29.5031	2.0543	
480.72	464.9524	15.7351	3.4018	
479.14	463.4242	7.2308	6.5743	
472.47	456.9730	139.5987	1.3796	δ HOCC (19)
464.31	449.0806	11.2917	3.726	
435.93	421.6315	5.4961	2.3033	
424.71	410.7795	0.9915	2.2714	
416.11	402.4616	0.2766	3.1135	
408.57	395.1689	1.3382	0.8722	
386.48	373.8035	5.6734	1.1995	
383.28	370.7084	5.3109	1.1559	
374.85	362.5549	0.9993	0.5542	
368.54	356.4519	2.2063	1.1443	
351.3	339.7774	6.8936	1.7143	
330.57	319.7273	5.6008	0.952	β HCC (15)
320.73	310.2101	4.2792	1.8641	
313.35	303.0721	5.0272	0.6383	
305.96	295.9245	4.9935	1.6967	
298.28	288.4964	7.2285	0.4919	
294.96	285.2853	37.496	0.7818	δ HCOH (11)
278.2	269.0750	4.1445	2.8998	
272.4	263.4653	65.1	2.0905	δ HCOH (13)
269.46	260.6217	9.2387	1.9161	
260.27	251.7331	1.6293	0.5243	
248.83	240.6684	3.8725	0.6946	
243.7	235.7066	3.5296	0.822	
233.02	225.3769	2.3314	0.1344	δ HCCC (12)
222.7	215.3954	3.486	0.1852	
217.64	210.5014	1.0668	0.6864	
212.15	205.1915	4.1438	0.8046	
197.99	191.4959	5.3267	0.6484	
185.25	179.1738	3.8284	0.8087	δ HOCC (18)
176.42	170.6334	2.2875	0.5201	
156.83	151.6860	0.7338	0.8476	δ HOCC (15)
142.8	138.1162	0.4301	1.0645	
137.38	132.8739	0.2603	0.236	
129.38	125.1363	1.0033	1.0058	δ HCCC (16)
116.64	112.8142	0.359	0.8581	δ HCCC (10)
112.36	108.6746	1.4665	0.7454	
97.84	94.6308	4.2624	0.761	
81.17	78.5076	2.7018	0.2181	
70.16	67.8588	3.8421	0.8586	
63.2	61.1270	3.2386	0.5827	
58.8	56.8714	0.4756	0.1693	
44.2	42.7502	1.6779	1.4354	
31.31	30.2830	0.0435	0.7544	
21.3	20.6014	0.8418	0.2563	
12.3	11.8966	0.5955	0.4571	

a Scaling factor: 0.9672 (6-311G(d)/B3LYP)

Table S2: PED Analysis–Cation State: This table presents the Potential Energy Distribution (PED) of vibrational modes for the cation state of Withaferin A, computed at the B3LYP/6-311G(d) level. Mode assignments and dominant internal coordinate contributions are included. Modes without assignments have low or distributed contributions, indicating mixed, delocalized, or weak vibrational motions. Here, ν denotes stretching vibrations, β denotes bending vibrations, and δ denotes torsional vibrations. Vibrational modes with less than 10% PED contribution aren't assigned.

Calculated Frequencies (cm^{-1})		IR Intensity	Raman Intensity	Assignment
Unscaled	Scaled[a]			
3780.05	3656.06436	106.9096	2312.6387	β HCC (99)
3731.91	3609.503352	1392.8254	5188.4694	δ HOCC (94)
3204.93	3099.808296	11.9499	3093.517	ν CH (86) + ν CH (11)
3183.68	3079.255296	16.9674	876.9813	ν CH (11) + ν CH (87)
3155.71	3052.202712	6.0003	7990.2691	ν CH (96)
3155.55	3052.04796	1.7994	483.5462	ν CH (71)
3149.4	3046.09968	2.3055	107.7764	ν CH (77)
3148.95	3045.66444	2.6266	305.655	ν CH (87)
3138.16	3035.228352	6.17	106.8356	ν CH (92)
3130.05	3027.38436	28.9815	2320.6216	ν CH (78) + ν CH (13)
3122.66	3020.236752	9.3195	151.1676	ν CH (83)
3121.95	3019.55004	13.0763	359.4606	ν CH (77)
3116.18	3013.969296	23.3137	39.8566	ν CH (87)
3112.83	3010.729176	21.2088	142.0748	ν CH (85)
3107.7	3005.76744	15.8965	185.9051	ν CH (89)
3103.69	3001.888968	19.7582	205.2973	ν CH (87)
3101.16	2999.441952	32.023	1115.3756	ν CH (82)
3087.06	2985.804432	15.3462	58.8492	ν CH (90)
3078.83	2977.844376	19.4217	121.2902	ν CH (88)
3074.93	2974.072296	22.7572	118.4994	ν CH (80)
3069.46	2968.781712	3.1656	710.7045	ν CH (97)
3060.66	2960.270352	27.5866	175.45	ν CH (78)
3056.55	2956.29516	39.2651	691.3011	ν CH (95)
3055.83	2955.598776	26.7191	50.6085	ν CH (12) + ν CH (77)
3052.62	2952.494064	250.7096	4073.8064	ν CH (13) + ν CH (80)
3050.8	2950.73376	35.3577	53.8684	ν CH (79)
3050.26	2950.211472	4.5492	562.53	ν CH (82)
3049.52	2949.495744	6.3382	67.9365	ν CH (79)
3040.2	2940.48144	7.0822	120.823	ν CH (74)
3037.85	2938.20852	2.8943	153.5535	ν CH (76) + ν CH (11)
3030.13	2930.741736	2.626	4003.808	ν CH (10) + ν CH (80)
3027.77	2928.459144	5.057	1759.0311	ν CH (79)
3022.66	2923.516752	21.0582	57.752	ν CH (81)
3014.2	2915.33424	25.9823	574.4863	ν CH (84)
3011.24	2912.471328	53.5849	2133.7344	ν CH (64) + ν CH (10)
2995.77	2897.508744	6.7969	82.3036	ν CH (83)
2981.73	2883.929256	4.3648	161.9262	ν CH (16) + ν CH (74)
2966.89	2869.576008	11.3897	925.6125	ν CH (96)
1724.92	1668.342624	185.3367	3141.7142	β HCC (12) + ν CC (24) + ν CC (13)
1700.27	1644.501144	230.9737	949.0805	ν CC (69)
1652.51	1598.307672	104.4208	2584.6491	ν CC (59)
1576.46	1524.752112	1310.3637	7666.2632	ν CC (20)
1541.6	1491.03552	16.8479	28.5963	δ HCCC (39)
1539.88	1489.371936	4.5901	14.6555	β HCH (40)
1530.45	1480.25124	0.2599	153.1181	β HCH (45)
1525.37	1475.337864	2.0372	94.6452	β HCC (39)
1521.27	1471.372344	21.3681	138.1662	β HCH (63)
1517.89	1468.103208	2.0403	3.909	β HCC (57)
1508.71	1459.224312	8.8839	38.7797	
1504.48	1455.133056	3.2613	269.1461	β HCH (46)
1499.55	1450.36476	243.2401	94.3475	β HCH (52)
1498.79	1449.629688	15.5118	224.0278	β HCH (58)

Table S2 continued from previous page

1491.89	1442.956008	10.711	44.3359	β HCH (18)
1491.33	1442.414376	3.8165	38.4909	β HCH (10) + β HCH (59)
1490.8	1441.90176	8.5042	44.0829	δ HCOC (24)
1476.35	1427.92572	19.4542	535.8411	β HCH (16)
1475.35	1426.95852	1.4197	339.0231	δ HCOC (11) + β HCO (23)
1454.04	1406.347488	238.4261	8388.4484	ν CC (30)
1450.71	1403.126712	17.8116	30.7532	
1444.5	1397.1204	13.6231	479.7604	ν OH (11) + β HCO (11)
1437.95	1390.78524	11.7002	26.2785	β HCC (33)
1432.08	1385.107776	41.3141	201.5495	β HOC (35)
1425.51	1378.753272	15.4033	23.6027	β HCH (27)
1422.56	1375.900032	108.8872	1802.1916	ν OH (10) + β HCH (30)
1410.43	1364.167896	159.9024	404.0172	
1407.04	1360.889088	8.1159	258.3502	β HCC (10)
1398.35	1352.48412	5.5605	25.2465	
1394.71	1348.963512	43.643	468.6848	δ HCCC (17)
1390.7	1345.08504	18.2106	153.4056	β HCH (18)
1387.35	1341.84492	4.9486	2832.0653	
1381.78	1336.457616	9.5288	1360.875	β HCC (16)
1380.52	1335.238944	42.3804	1208.323	
1372.55	1327.53036	9.8053	71.8876	β HCC (16)
1369.96	1325.025312	57.5624	4151.0962	ν CC (19)
1356.54	1312.045488	48.4523	458.7718	β HCO (11)
1354.15	1309.73388	14.6074	1013.3781	
1350.82	1306.513104	5.1152	166.3576	δ HCCC (10)
1349.21	1304.955912	23.719	523.2104	β HCC (11)
1339.72	1295.777184	24.4147	511.003	
1332.1	1288.40712	4.201	420.2488	
1322.36	1278.986592	7.6434	3801.0119	
1314.41	1271.297352	9.3702	49.4354	
1311.38	1268.366736	63.8678	2678.9472	
1306.13	1263.288936	2.9232	201.1154	
1298.08	1255.502976	11.6757	297.7833	
1286.5	1244.3028	42.7392	207.6545	
1280.45	1238.45124	29.3496	167.7465	
1268.17	1226.574024	53.609	869.6847	β HCC (11)
1260.81	1219.455432	13.2086	69.8387	
1249.79	1208.796888	7.4014	446.3852	
1247.68	1206.756096	166.7754	96.1244	δ HCCC (28)
1239.92	1199.250624	10.6632	262.7613	β HCC (10)
1223.6	1183.46592	12.1011	2267.151	
1220.37	1180.341864	46.1139	1385.9375	
1214.38	1174.548336	26.8164	728.6805	
1209.92	1170.234624	5.1185	305.6996	
1202.73	1163.280456	381.3548	1415.839	
1186.31	1147.399032	26.3819	900.3207	
1170.65	1132.25268	225.0048	541.4813	
1161.86	1123.750992	66.6718	40.1674	
1152.68	1114.872096	2.896	605.1476	β HCO (10)
1145.03	1107.473016	31.5107	84.0308	
1136.67	1099.387224	111.6477	640.3805	
1130.79	1093.700088	72.3045	1721.1028	
1128.22	1091.214384	89.2069	896.4807	
1108.87	1072.499064	210.4822	57.154	
1102.06	1065.912432	443.9769	613.0361	β HCC (24)
1090.77	1054.992744	9.8052	194.8554	
1087.69	1052.013768	538.9675	12627.2662	ν CC (11)
1081.61	1046.133192	52.65	553.0141	
1077.04	1041.713088	2.1015	831.5218	
1074.27	1039.033944	5.9515	195.9474	ν CC (16)
1066.75	1031.7606	2.7515	1650.4373	ν CC (25)
1063.31	1028.433432	7.7793	507.8014	β HCC (11)
1056.86	1022.194992	173.1692	320.3501	ν CC (24)
1053.56	1019.003232	224.5938	462.0983	ν CC (30)
1049.53	1015.105416	161.2794	234.2457	ν CC (26)
1041.88	1007.706336	76.5922	594.3805	δ HCCC (21)
1027.29	993.594888	307.6977	833.9241	

Table S2 continued from previous page

1024.42	990.819024	191.2118	188.7407	ν CC (21)
1011.76	978.574272	45.7304	1171.357	ν CC (30)
1005.06	972.094032	35.8644	69.7198	ν CC (22)
1003.99	971.059128	30.8743	2726.1975	
1000.19	967.383768	98.4861	2134.3792	ν CC (16)
993.97	961.367784	348.3425	2277.6435	ν CC (18)
974.94	942.961968	39.6601	1829.16	ν CC (23)
966.91	935.195352	38.0731	421.1377	
964.99	933.338328	217.3729	1101.5741	ν CC (12)
958.96	927.506112	29.2174	45.6842	ν CC (10)
957.13	925.736136	176.1132	592.8281	ν CC (18)
948.16	917.060352	7.2596	229.7441	
945.36	914.352192	178.3024	1678.0568	β HCO (10)
927.92	897.484224	8.9706	21.4312	ν CC (17)
926.38	895.994736	194.7995	88.3191	
906.98	877.231056	147.6852	61.152	ν CC (20)
898.12	868.661664	18.6003	1535.3661	δ HCCC (16)
871.06	842.489232	43.7471	176.3016	β HCH (11)
860.6	832.37232	161.2458	1062.0272	
853.75	825.747	268.1912	893.295	
842.8	815.15616	105.2571	60.3654	δ HCCC (18)
829.18	801.982896	11.7385	3758.679	
797.29	771.138888	6.8312	144.7211	ν CC (27)
793.15	767.13468	81.8936	171.1976	ν CC (16)
780.01	754.425672	6.6739	372.5449	δ HCCC (10)
759.79	734.868888	182.1449	1387.9583	δ HCCC (17)
744.97	720.534984	0.602	219.5438	β HCC (10)
731.65	707.65188	9.603	25.4888	
716.5	692.9988	261.5207	403.3922	ν CC (12)
711.66	688.317552	799.2063	1758.9455	
706.07	682.910904	58.6262	97.3495	
663.65	641.88228	16.0951	210.6462	β HCC (11)
638.83	617.876376	39.782	653.208	
626.9	606.33768	3.2332	337.8372	
614.92	594.750624	24.0369	515.9626	
589.57	570.232104	30.8859	755.2279	
577.8	558.84816	1.4392	96.6583	
568.85	550.19172	198.5699	87.6864	
557.49	539.204328	31.3057	700.0992	
547.4	529.44528	12.5295	210.2169	
539.36	521.668992	61.29	1289.9131	
537.02	519.405744	8.5908	50.3303	
507.29	490.650888	2.697	365.0519	
488.07	472.061304	9.7443	276.2701	δ HCCC (17)
480.09	464.343048	21.9766	156.9721	
473.98	458.433456	243.4722	95.5107	
464.92	449.670624	34.2644	86.4274	β HCH (14)
439.13	424.726536	916.5919	103.5812	
427.96	413.922912	455.27	62.1112	
421.74	407.906928	167.9815	203.0811	β HCC (12)
415.56	401.929632	48.9321	22.0558	
401.76	388.582272	64.6166	98.8011	
393.25	380.3514	189.3761	345.762	
381.19	368.686968	25.9027	85.447	
371.03	358.860216	3.1918	18.5902	β HCC (22)
363.74	351.809328	3.7065	66.2339	
356.58	344.884176	205.8386	3037.721	
344.98	333.664656	6.0343	14.8528	
329.4	318.59568	28.5772	64.339	
315.97	305.606184	40.119	100.0238	β HCC (15)
310.74	300.547728	12.9255	495.9807	
306.26	296.214672	5.1333	80.8247	
301.21	291.330312	2.7206	38.2061	
297.74	287.974128	3.2402	23.3678	
276.61	267.537192	7.0367	7.7278	
265.33	256.627176	9.9146	1061.057	δ HOCC (16)
262.91	254.286552	1.9675	165.9817	

Table S2 continued from previous page

252.7	244.41144	9.0917	108.7245	
242.87	234.903864	23.4408	113.8136	δ HCCC (12)
233.06	225.415632	7.1835	9.6157	
226.38	218.954736	0.2142	8.6348	
218.86	211.681392	14.8626	74.681	
213.61	206.603592	26.6637	56.5061	
194.5	188.1204	8.3452	203.7414	
183.2	177.19104	14.2429	403.3596	
175.31	169.559832	2.6139	119.6814	
151.53	146.559816	22.7717	106.7771	
139.56	134.982432	12.2409	34.0879	δ HOCC (16)
136.62	132.138864	0.8552	59.1737	δ HOCC (12)
127.85	123.65652	0.885	6.8855	δ HOCC (11)
119.23	115.319256	12.2005	30.8613	
113.35	109.63212	3.0357	161.2626	
92.21	89.185512	7.8183	75.3378	
81.74	79.058928	2.7532	92.5675	
79.68	77.066496	9.4772	21.3699	
66.28	64.106016	3.9577	98.8218	
59.51	57.558072	0.1997	18.6433	
43.57	42.140904	0.1233	105.7205	
26.94	26.056368	0.7784	36.5708	β HOC (12)
22.75	22.0038	0.7654	24.4843	
11.87	11.480664	0.6601	12.9869	

a Scaling factor: 0.9672 (6-311G(d)/B3LYP)

Table S3: PED Analysis–Anion State: This table presents the Potential Energy Distribution (PED) of vibrational modes for the anion state of Withaferin A, computed at the B3LYP/6-311G(d) level. Mode assignments and dominant internal coordinate contributions are included. Modes without assignments have low or distributed contributions, indicating mixed, delocalized, or weak vibrational motions. Here, ν denotes stretching vibrations, β denotes bending vibrations, and δ denotes torsional vibrations. Vibrational modes with less than 10% PED contribution aren't assigned.

frequencies calculated (cm^{-1})		IR intensity	Raman Intensity	Assignment
Unscaled	Scaled[a]			
3733.46	3611.002512	122.8151	29117.8872	ν OH (100)
3731.75	3609.3486	7.0552	2490.1262	ν OH (100)
3178.79	3074.525688	14.7775	7893.8547	ν CH (74) + ν CH (26)
3141.57	3038.526504	9.4216	8.3367	ν CH (77) + ν CH (11)
3136.28	3033.410016	182.3504	1059.4557	ν CH (26) + ν CH (73)
3130.27	3027.597144	10.1156	18.3719	ν CH (77)
3125.85	3023.32212	44.4101	314.6696	ν CH (92)
3119.62	3017.296464	12.1877	88.904	ν CH (80)
3118.08	3015.806976	71.1665	181.8534	ν CH (71)
3115.98	3013.775856	14.0105	55.129	ν CH (74)
3114.31	3012.160632	23.2118	10.9143	ν CH (78)
3102.89	3001.115208	3.1037	10.4732	ν CH (80)
3095.79	2994.248088	45.078	60.4868	ν CH (87)
3091.56	2990.156832	36.7002	26.8954	ν CH (65) + ν CH (14)
3090.67	2989.296024	3.6788	65.306	ν CH (17) + ν CH (76)
3088.07	2986.781304	23.7294	167.8039	ν CH (98)
3075.67	2974.788024	14.7381	57.54	ν CH (88)
3058.55	2958.22956	81.2815	41.2108	ν CH (70)
3056.21	2955.966312	9.7673	67.9129	ν CH (78)
3046.89	2946.952008	124.4988	181.8661	ν CH (83)
3045.14	2945.259408	60.6798	310.0842	ν CH (84)
3036.01	2936.428872	40.199	73.2144	ν CH (74)

Table S3 continued from previous page

3033.59	2934.088248	131.8526	399.6343	ν CH (85)
3031.9	2932.45368	60.2233	31.5423	ν CH (76)
3031.43	2931.999096	19.1738	71.7531	ν CH (80)
3028.39	2929.058808	5.0876	57.9587	ν CH (72)
3024.66	2925.451152	2.0253	28.2755	ν CH (81)
3015.9	2916.97848	19.4361	48.9574	ν CH (79)
3011.82	2913.032304	2.853	27.3274	ν CH (89)
3004.92	2906.358624	25.7612	45.4537	ν CH (72)
2993.4	2895.21648	42.5231	152.3395	ν CH (69)
2989.89	2891.821608	55.4671	2397.6387	ν CH (99)
2981.28	2883.494016	17.596	50.9455	ν CH (11) + ν CH (68)
2970.34	2872.912848	1198.0695	11464.7901	ν CH (91)
2965.66	2868.386352	652.4647	7014.6689	ν CH (97)
2961.73	2864.585256	5.6148	7.5996	ν CH (82)
2944.82	2848.229904	375.6086	1924.7421	ν CH (87)
2871.05	2776.87956	756.2648	8036.0456	ν CH (98)
1706.66	1650.681552	2535.5322	4778.2842	ν CC (20)
1624.36	1571.080992	37.7618	2394.2296	ν CC (46)
1569.91	1518.416952	10669.0517	589.7249	ν CC (17)
1541.96	1491.383712	7.0757	5.3273	β HCH (30)
1538.14	1487.689008	227.1674	46.3621	δ HCCC (26)
1536.78	1486.373616	657.5098	29.4973	β HCH (37)
1531.44	1481.208768	0.3916	2.1423	β HCH (44)
1526.56	1476.488832	105.3899	47.1994	β HCC (45)
1522.14	1472.213808	12.2259	24.2449	β HCH (56)
1520.47	1470.598584	29.4751	737.6498	β HCC (32)
1518.17	1468.374024	113.1872	2422.8899	ν CC (14)
1510.2	1460.66544	4.3483	116.0046	β HCH (46)
1505.72	1456.332384	11.6873	311.86	β HCH (44)
1502.05	1452.78276	5.5079	5.7424	β HCH (58)
1498.38	1449.233136	229.2347	730.6335	β HCH (46)
1497.88	1448.749536	34.5389	96.8954	β HCH (50)
1495.64	1446.583008	5.3218	88.8244	δ HCOC (19)
1487.41	1438.622952	153.2971	322.5708	β HCH (12) + β HCO (21) + β HCC (11)
1477.39	1428.931608	186.3777	227.5396	β HCO (11)
1476.29	1427.867688	225.6104	336.0606	ν CC (14)
1456.99	1409.200728	30.6457	3680.7964	β HCO (18)
1447.26	1399.789872	0.6686	10.4741	β HCO (17)
1437.56	1390.408032	21.2772	434.3621	β HCC (13)
1433.36	1386.345792	7.8	10.2989	β HOC (35)
1429.22	1382.341584	7.8107	8.789	β HCH (17)
1420.35	1373.76252	745.5646	1102.2812	β HCH (31)
1413.66	1367.291952	6.2287	21.2036	β HCH (25)
1406.96	1360.811712	4.4445	5.2043	β HCC (13)
1405.71	1359.602712	5.0554	4.9784	
1403.89	1357.842408	16.1071	30.2791	δ HCCC (19)
1402.42	1356.420624	208.3044	21.7156	β HCH (23)
1393.31	1347.609432	339.3947	20.5271	
1383.14	1337.773008	75.3507	56.961	β HCC (17)
1381.11	1335.809592	26.3828	12.4464	
1374.34	1329.261648	23.0424	125.9559	β HCC (14)
1366.21	1321.398312	101.1835	131.4559	
1359.05	1314.47316	24.268	55.2999	
1357.93	1313.389896	150.6724	168.3426	β HCC (12)
1354.52	1310.091744	4.6995	11.9114	β HCC (42)
1350.05	1305.76836	25.4141	87.5391	
1337.55	1293.67836	25.3518	16.3371	β HCC (12)
1333.85	1290.09972	30.2081	4.3565	β HCC (14)
1326.88	1283.358336	150.5625	78.9979	β HCC (19)
1323.39	1279.982808	26.7113	6.3784	β HCC (24)
1321.06	1277.729232	122.9405	40.7465	β HCO (19)
1313.03	1269.962616	1551.8557	399.9457	
1299.03	1256.421816	273.6235	6.9546	
1298.36	1255.773792	21.3775	4.4402	β HCO (16)
1286.11	1243.925592	34.8456	41.9771	
1266.37	1224.833064	35.77	141.2454	β HCC (12)
1263.01	1221.583272	66.8866	506.1553	

Table S3 continued from previous page

1258.02	1216.756944	131.164	403.869	
1251.73	1210.673256	178.0312	237.0696	
1248.25	1207.3074	51.6114	99.5376	
1245.17	1204.328424	344.7297	62.9738	
1242.83	1202.065176	160.4662	25.5676	
1226.42	1186.193424	59.8223	4.3009	
1217.37	1177.440264	1554.676	61.6229	
1214.38	1174.548336	835.3461	41.9989	β HCC (16)
1199.13	1159.798536	644.3553	1826.2415	ν CC (30)
1191.08	1152.012576	317.2956	18.228	
1179.11	1140.435192	168.4968	305.3863	
1168.55	1130.22156	36.0032	673.0319	
1156.05	1118.13156	197.233	220.5147	
1149.61	1111.902792	318.1158	46.7887	β HCC (12)
1145.97	1108.382184	171.9468	84.3907	
1135.17	1097.936424	9.1387	101.7602	
1129.46	1092.413712	18.1245	79.2348	
1117.9	1081.23288	1345.0697	94.5564	
1109.45	1073.06004	299.2106	47.524	β HCC (12)
1105.82	1069.549104	7.8125	49.3858	ν CC (26)
1094.76	1058.851872	6.6148	29.5625	
1087.01	1051.356072	57.4883	75.9379	ν CC (33)
1080.02	1044.595344	379.2899	9.98	ν CC (11)
1079.09	1043.695848	196.4731	28.0906	ν CC (24)
1070.05	1034.95236	80.8624	8.393	
1063.65	1028.76228	2079.7716	163.5891	ν CC (27)
1059.5	1024.7484	2211.7648	40.2754	ν CC (34)
1052.24	1017.726528	21.8536	21.2111	ν CC (23)
1041.21	1007.058312	167.1719	234.64	δ HCCC (16)
1031.38	997.550736	114.6581	15.9559	ν CC (33)
1025.22	991.592784	28.4093	351.9947	ν CC (25)
1018.68	985.267296	86.2086	145.5588	ν CC (27)
1015.37	982.065864	38.4794	80.3211	ν CC (28)
1006.82	973.796304	20.9761	103.0743	ν CC (24)
1002.87	969.975864	54.0738	297.8683	ν CC (22)
991.11	958.601592	27.4208	99.1163	ν CC (24)
982.64	950.409408	185.7428	107.9039	ν CC (22)
970.6	938.76432	58.3498	63.2835	
969.23	937.439256	846.383	113.1819	ν CC (24)
966.93	935.214696	36.9989	57.8564	ν CC (29)
952.63	921.383736	4.8101	5.2928	
945.41	914.400552	155.3239	10.3953	β HCH (12) + ν CC (30)
934.68	904.022496	29.5971	43.748	ν CC (24)
925.44	895.085568	91.4498	31.9615	ν CC (24)
910.9	881.02248	80.5057	13.3272	ν CC (20)
906.3	876.57336	57.7383	19.2206	ν CC (22)
880.44	851.561568	221.1125	63.4443	δ HCCC (22)
875.14	846.435408	10.84	36.8858	ν CC (27)
873.71	845.052312	134.52	156.9833	
852	824.0544	0.8885	4.3457	
841.83	814.217976	3.5034	8.2325	
820.85	793.92612	39.4357	17.7088	
795.9	769.79448	8.6407	22.8544	ν CC (15)
790.35	764.42652	9.0318	25.8733	ν CC (19)
765.47	740.362584	2.2655	12.9365	
761.18	736.213296	143.3571	21.4749	
735.61	711.481992	22.1017	41.4484	
723.81	700.069032	9.3461	46.4845	ν CC (31)
708.65	685.40628	17.3315	19.2421	ν CC (13)
702.44	679.399968	82.8214	29.0173	
692.63	669.911736	427.5133	14.6774	
667.49	645.596328	35.1117	120.7182	
644.74	623.592528	36.3295	110.3389	
631.13	610.428936	5.3189	13.2974	
612.23	592.148856	9.7697	10.2348	
596.28	576.722016	193.5174	79.2615	β HCC (12)
587.04	567.785088	14.781	82.401	β HOC (24)

Table S3 continued from previous page

566.42	547.841424	189.9501	49.3883	
552.15	534.03948	30.696	9.9767	
544.4	526.54368	145.3695	75.2636	β HCC (17)
530.68	513.273696	79.5353	44.6534	
526.23	508.969656	393.8138	146.1539	β HCC (10)
510.23	493.494456	14.5372	73.1237	β HCC (15)
493.87	477.671064	35.3085	73.7679	
475.69	460.087368	0.5216	15.2386	
473.32	457.795104	84.4286	86.0518	
470.75	455.3094	40.7803	176.4824	δ HCCC (21)
460.28	445.182816	15.9589	57.1307	
430.95	416.81484	57.3694	5.0799	β HCC (20)
422.05	408.20676	31.4972	1.798	
410.35	396.89052	89.5469	27.0411	
389.68	376.898496	19.2791	108.7298	β HCC (13)
382.24	369.702528	1.1558	62.0697	
380.39	367.913208	26.2092	17.7333	
373.71	361.452312	1.1218	33.9465	β HCC (15)
368.16	356.084352	0.4062	10.3084	
349.76	338.287872	2.9068	38.7526	β HOC (13)
336.24	325.211328	1.5966	2.9737	β HCO (18)
326.61	315.897192	54.6814	26.9336	
322.45	311.87364	34.5376	68.265	β HCC (22)
312.57	302.317704	98.3801	22.716	δ HCCC (11)
297.92	288.148224	5.7077	2.4995	
295.85	286.14612	30.9795	2.0081	
282.8	273.52416	77.201	34.4521	
267.61	258.832392	39.9514	12.3664	δ HCOH (10)
260.64	252.091008	15.203	20.7425	δ HOCC (11)
253.05	244.74996	26.3257	282.7967	δ HCOH (16)
249.92	241.722624	48.9367	387.8363	
243.06	235.087632	5.4508	7.7449	δ HCCC (19)
234.42	226.731024	9.2266	4.7603	δ HCOH (11) + δ HCCC (11)
222.14	214.853808	5.471	6.3359	
217.84	210.694848	0.2149	3.1561	
200.19	193.623768	6.2315	8.6421	δ HCCC (14)
195.31	188.903832	1.8631	16.9746	
185.06	178.990032	0.6433	5.5805	δ HOCC (12)
178.06	172.219632	6.3503	57.7962	
152.77	147.759144	5.464	39.3099	
142.28	137.613216	18.2848	2.2722	
130.55	126.26796	0.5601	8.1849	δ HCCC (10)
128.23	124.024056	1.6774	15.8539	δ HOCC (10) + δ HOCC (12)
116.54	112.717488	1.607	127.374	
103	99.6216	2.6574	32.1808	
92.49	89.456328	5.2374	125.1006	
79.8	77.18256	17.2561	2.2828	δ HOCC (11)
73.59	71.176248	0.9761	25.3564	δ HCCC (12)
72.84	70.450848	2.5316	123.9488	
61.72	59.695584	1.4251	3.2367	
45.86	44.355792	0.1288	42.7784	
35.38	34.219536	0.1625	13.3543	
24.69	23.880168	0.6103	9.3719	
18.02	17.428944	1.827	10.6025	

a Scaling factor: 0.9672 (6-311G(d)/B3LYP)

Table S4: NBO Analysis–Neutral State: This table presents key donor–acceptor interactions in Withaferin A's neutral state. Stabilization energies [$E^{(2)}$], energy differences (E_j–E_i), and Fock matrix elements [F(i,j)] were calculated at the B3LYP/6-311G(d) level.

Donor(i)	Acceptor(j)	$E^{(2)}$ [kcal/mol]	E_j-E_i [a.u.]	F(i,j) [a.u.]
BD(2)C1-C2	BD*(2)C3-O30	19.21	0.31	0.069
BD(1)C1-H36	BD*(1)C2-C3	5.27	0.98	0.065
BD(1)C2-H35	BD*(1)C1-C5	5.14	0.93	0.062
BD(2)C3-O30	BD*(2)C1-C2	5.29	0.42	0.043
BD(1)C6-O29	BD*(1)C9-O29	5.65	0.93	0.065
BD(1)C9-O29	BD*(1)C6-O29	5.85	0.9	0.065
BD(1)C10-H43	BD*(1)C9-O29	5.75	0.73	0.058
BD(1)C14-H51	BD*(1)C13-C32	5.21	0.83	0.059
BD(1)C21-C22	BD*(1)C25-C27	5.23	1.04	0.066
BD(1)C21-H66	BD*(2)C22-C25	5.06	0.56	0.048
BD(2)C22-C25	BD*(2)C24-O33	19.75	0.32	0.072
BD(1)C24-C25	BD*(1)C22-C26	5.44	1.08	0.068
BD(1)C25-C27	BD*(1)C22-C25	5.13	1.29	0.073
BD(1)C26-H70	BD*(2)C22-C25	5.47	0.56	0.05
CR(1)O30	RY*(1)C3	6.62	19.63	0.323
CR(1)O33	RY*(1)C24	6.62	19.72	0.324
LP(1)O23	BD*(1)C24-C25	6	0.97	0.069
LP(2)O23	BD*(1)C20-H64	6.12	0.72	0.062
LP(2)O23	BD*(2)C24-O33	34.66	0.36	0.101
LP(2)O28	BD*(1)C5-C6	6.57	0.68	0.06
LP(2)O28	BD*(1)C5-H37	6.68	0.69	0.061
LP(2)O29	BD*(1)C4-C6	5.07	0.68	0.053
LP(2)O29	BD*(1)C9-H42	5.29	0.72	0.056
LP(1)O30	RY*(1)C3	15.04	1.46	0.132
LP(2)O30	BD*(1)C2-C3	18.43	0.71	0.104
LP(2)O30	BD*(1)C3-C4	20.46	0.63	0.102
LP(1)O33	RY*(1)C24	15.52	1.56	0.139
LP(2)O33	BD*(1)O23-C24	31.7	0.62	0.127
LP(2)O33	BD*(1)C24-C25	17.07	0.68	0.098
LP(2)O34	BD*(1)C27-H67	5.6	0.71	0.057
LP(2)O34	BD*(1)C27-H68	6.53	0.68	0.06
BD*(2)C3-O30	BD*(2)C1-C2	26.99	0.02	0.069
BD*(2)C24-O33	BD*(2)C22-C25	61.08	0.02	0.07

Table S5: NBO Analysis–Cation State: This table presents key donor–acceptor interactions in Withaferin A's neutral state. Stabilization energies [$E^{(2)}$], energy differences (E_j–E_i), and Fock matrix elements [F(i,j)] were calculated at the B3LYP/6-311G(d) level.

Donor(i)	Acceptor(j)	$E^{(2)}$ [kcal/mol]	E_j-E_i [a.u.]	F(i,j) [a.u.]
BD(2)C1-C2	BD*(2)C3-O30	12.89	0.29	0.077
BD(2)C22-C25	BD*(2)C24-O33	9.52	0.32	0.071
LP(2)O23	BD*(2)C24-O33	18.8	0.36	0.105
LP(1)O30	RY*(1)C3	7.33	1.49	0.132
LP(2)O30	BD*(1)C2-C3	8.57	0.77	0.104
LP(2)O30	BD*(1)C3-C4	9.98	0.64	0.101
LP(1)O33	RY*(1)C24	7.44	1.58	0.137
LP(2)O33	BD*(1)O23-C24	15.1	0.65	0.127
LP(2)O33	BD*(1)C24-C25	8.31	0.68	0.097
BD*(2)C3-O30	BD*(2)C1-C2	8.45	0.04	0.07
BD*(2)C24-O33	BD*(2)C22-C25	37.32	0.01	0.069

Table S6: NBO Analysis–Anion State: This table presents key donor–acceptor interactions in Withaferin A's anion state. Stabilization energies [$E^{(2)}$], energy differences (E_j–E_i), and Fock matrix elements [F(i,j)] were calculated at the B3LYP/6-311G(d) level.

Donor NBO(i)	Acceptor NBO(j)	$E^{(2)}$ [kcal/mol]	E_j-E_j [a.u]	F(i,j) [a.u]
BD(2)C22-C25	BD*(2)C24-O33	9.42	0.3	0.072
LP(1)C3	BD*(2)C1-C2	47.44	0.14	0.113
LP(2)O23	BD*(2)C24-O33	14.61	0.34	0.096
LP(1)O30	RY*(1)C3	6.67	1.48	0.125
LP(2)O30	BD*(1)C2-C3	7.27	0.77	0.096
LP(2)O30	BD*(1)C3-C4	9.74	0.6	0.098
LP(1)O33	RY*(1)C24	7.59	1.53	0.136
LP(2)O33	BD*(1)O23-C24	15.7	0.6	0.124
LP(2)O33	BD*(1)C24-C25	7.64	0.71	0.095
BD*(2)C24-O33	BD*(2)C22-C25	167.17	0.01	0.085

Table S7: UV-VIS data: Calculated UV-VIS absorption wavelengths (nm), oscillator strengths (f), band gaps (eV), and major electronic transitions with percentage contributions for neutral, cation, and anion states of Withaferin A. Electron excitations are indicated between molecular orbitals (e.g., HOMO → LUMO). Calculations performed using TD-DFT with the 6-311G(d) basis set.

	Wavelength (nm)	Oscillator strength(f)	Bandgap (eV)	Major contributions
Neutral	337.94	0.002	3.6688	HOMO→LUMO (95.13%)
	273.25	0.0228	4.5374	H-2→LUMO (92.88%),<br>H-1→LUMO (2.83%)
	255.16	0.0086	4.8591	H-5→L+1 (13.42%), H-4→L+1 (12.43%),<br>H-3→L+1 (39.31%), H-1→L+1(27.90%)
Cation	5245.11	0.0162	0.2364	β H-1→β LUMO (113.37%),<br>β HOMO→β LUMO (226.74%),<br>β HOMO←β LUMO (142%)
	4189.41	0.044	0.2959	β H-2→β LUMO (3.04%),<br>β H-1→β LUMO (84.39%),<br>β HOMO→β LUMO (266.08%),<br>β HOMO←β LUMO (155.82%)
	2278.52	0.0031	0.5441	β H-4→β LUMO (2.94%),<br>β H-3→β LUMO (179.28%),<br>β H-2→β LUMO (16.68%)
Anion	20730.05	0.0038	0.0598	α HOMO→ α LUMO (1831.82%),<br>α HOMO←α LUMO (1631.54%)
	1641.55	0.0139	0.7553	α HOMO→ α L+1 (196.21%),<br>α HOMO→ α L+2 (3.81%)
	1285.5	0.0084	0.9645	α HOMO→ α L+1 (3.85%),<br>α HOMO→ α L+2 (195.90%)

Table S8: Fukui Function values for Withaferin A: Fukui function values for electrophilic attack (f^-), nucleophilic attack (f^+), and average (f^0), along with the dual descriptor for atoms in Withaferin A. The data were computed using B3LYP/6-311G(d) level of theory. Positive dual descriptor values indicate sites more prone to nucleophilic attack, while negative values correspond to electrophilic susceptibility.

Atoms	Sites	f^-	f^+	f^0	Dual Descriptor
C	1	0.063	0.1447	0.1039	0.0817
C	2	-0.0127	0.0385	0.0129	0.0258
C	3	0.0193	0.1328	0.076	0.1136
C	4	0.0431	-0.0132	0.015	-0.0299
C	5	-0.0082	-0.0117	0.01	0.0034
C	6	-0.0127	-0.0158	0.0143	0.0031
C	7	0.0001	0.0019	0.001	0.0019
C	8	0.0075	0.0002	0.0039	-0.0073
C	9	0.0074	0.0073	0.0073	-0.0002
C	10	-0.0049	-0.0049	0.0049	0
C	11	0.0047	-0.0008	0.0019	-0.0038
C	12	-0.0026	0.0006	0.001	-0.002
C	13	0.008	-0.0009	0.0035	-0.0071
C	14	0.0025	-0.0017	0.0004	-0.0007
C	15	-0.0012	-0.0008	0.001	-0.0004
C	16	-0.0006	0.0009	0.0001	0.0003
C	17	0.0081	-0.0006	0.0037	-0.0074
C	18	0.0032	-0.0013	0.0009	-0.0018
C	19	-0.0024	-0.0011	0.0017	-0.0013
C	20	0.0019	-0.002	0.0001	0.0002
C	21	-0.003	-0.0057	0.0043	0.0027
C	22	0.0268	0.0698	0.0483	0.0429
O	23	0.0323	0.0184	0.0254	-0.0139
C	24	0.0013	0.0395	0.0204	0.0382
C	25	0.0511	0.0431	0.0471	-0.008
C	26	-0.0046	-0.0083	0.0064	0.0036
C	27	0.0002	-0.0011	0.0004	0.0009
O	28	0.0105	0.0143	0.0124	0.0038
O	29	0.0457	0.0217	0.0337	-0.024
O	30	0.1682	0.1242	0.1462	-0.044
C	31	-0.0053	0.0006	0.0023	-0.0046
C	32	-0.0029	-0.001	0.002	-0.0019
O	33	0.0345	0.0537	0.0441	0.0192
O	34	0.1011	0.0065	0.0538	-0.0946
H	35	0.0233	0.0264	0.0248	0.0031
H	36	0.0204	0.0249	0.0227	0.0045
H	37	0.0181	0.0307	0.0244	0.0126
H	38	0.0097	0.015	0.0124	0.0053
H	39	0.0052	0.006	0.0056	0.0008
H	40	0.0132	0.0074	0.0103	-0.0058
H	41	0.0181	0.0189	0.0185	0.0008
H	42	0.0179	0.0102	0.014	-0.0076
H	43	0.0079	0.0063	0.0071	-0.0016
H	44	0.0138	0.0056	0.0097	-0.0082
H	45	0.0076	0.0054	0.0065	-0.0022
H	46	0.0004	-0.0135	0.0065	0.0131
H	47	0.0086	0.0045	0.0066	-0.0041
H	48	0.0054	0.0041	0.0047	-0.0013
H	49	0.0132	0.008	0.0106	-0.0052
H	50	0.0135	0.0134	0.0135	0
H	51	0.0052	0.0024	0.0038	-0.0028
H	52	0.0096	0.007	0.0083	-0.0026
H	53	0.0068	0.0042	0.0055	-0.0026
H	54	0.0071	0.0053	0.0062	-0.0017
H	55	0.0056	0.0052	0.0054	-0.0004
H	56	0.003	-0.0018	0.0006	-0.0012

Table S8 continued from previous page

H	57	0.0048	0.0023	0.0035	-0.0025
H	58	0.0026	0.0013	0.0019	-0.0014
H	59	0.0062	0.0025	0.0044	-0.0037
H	60	0.0049	0.0023	0.0036	-0.0026
H	61	0.0052	0.0031	0.0042	-0.002
H	62	0.006	0.0046	0.0053	-0.0014
H	63	0.0052	0.0036	0.0044	-0.0016
H	64	0.0085	0.0064	0.0075	-0.0021
H	65	0.0134	0.0157	0.0146	0.0023
H	66	0.0182	0.0203	0.0192	0.002
H	67	0.0023	0.0054	0.0039	0.0031
H	68	0.0387	0.0184	0.0286	-0.0203
H	69	0.0331	0.0171	0.0251	-0.016
H	70	0.0146	0.0217	0.0182	0.0072
H	71	0.0112	0.0146	0.0129	0.0034
H	72	0.0125	0.0174	0.0149	0.0049
